# Nucleosome compaction facilitates HP1γ binding to methylated H3K9

**DOI:** 10.1093/nar/gkv841

**Published:** 2015-08-28

**Authors:** Yuichi Mishima, Chanika D. Jayasinghe, Kai Lu, Junji Otani, Masahiro Shirakawa, Toru Kawakami, Hironobu Kimura, Hironobu Hojo, Peter Carlton, Shoji Tajima, Isao Suetake

**Affiliations:** 1 Laboratory of Epigenetics, Institute for Protein Research, Osaka University, Suita, Osaka 565-0871, Japan; 2 Institute for Integrated Cell-Material Sciences, Kyoto University, Kyoto 606-8501, Japan; 3 Department of Molecular Engineering, Graduate School of Engineering, Kyoto University, Nishikyo-ku, Kyoto 615-8510, Japan; 4 CREST, Japan Science and Technology Agency, Saitama 332-0012, Japan; 5 Laboratory of Organic Chemistry, Institute for Protein Research, Osaka University, Osaka 565-0871, Japan

## Abstract

The α, β and γ isoforms of mammalian heterochromatin protein 1 (HP1) selectively bind to methylated lysine 9 of histone H3 via their chromodomains. Although the phenotypes of HP1-knockout mice are distinct for each isoform, the molecular mechanisms underlying HP1 isoform-specific function remain elusive. In the present study, we found that in contrast to HP1α, HP1γ could not bind tri-methylated H3 lysine 9 in a reconstituted tetra-nucleosomes when the nucleosomes were in an uncompacted state. The hinge region connecting HP1's chromodomain and chromoshadow domain contributed to the distinct recognition of the nucleosomes by HP1α and HP1γ. HP1γ, but not HP1α, was strongly enhanced in selective binding to tri-methylated lysine 9 in histone H3 by the addition of Mg^2+^ or linker histone H1, which are known to induce compaction of nucleosomes. We propose that this novel property of HP1γ recognition of lysine 9 in the histone H3 tail in different nucleosome structures plays a role in reading the histone code.

## INTRODUCTION

In eukaryotes, there are broadly speaking two distinct states of chromatin: euchromatin, which is in a relaxed or extended state and generally transcriptionally active, and heterochromatin, which is in a condensed state and transcriptionally inactive (1). Methylation at lysine 9 of histone H3 (H3K9me) plays a crucial role in heterochromatinization (2). Among the three methylation states of H3K9, a high level of both di- (me2) and/or tri-methylation (me3) are found in transcriptionally silent genes (3,4), and especially, H3K9me3 in DAPI-dense heterochromatin regions (5).

A highly conserved chromatin-binding protein named heterochromatin protein 1 (HP1) or its orthologue exist in organisms ranging from yeast to human (6). HP1 possesses two conserved domains, the N-terminal chromodomain (CD), which selectively recognizes H3K9me2 and me3 (7), and the chromoshadow domain (CSD), through which HP1 forms dimers with other HP1 molecules or with other proteins (7), and regulates the binding activity of the CD (8). It was also reported that the CD is not only responsible for the recognition of H3K9me2 and me3 but also possesses the ability to dimerize, although with low affinity (9,10). These two highly conserved domains, the CD and CSD, are separated by a sequence called the hinge region (HR). As well as being divergent among HP1 isoforms, the HR sequence is proposed to be flexible and exposed to the molecular surface (7).

In mammals, three HP1 isoforms, α, β and γ, have been isolated (11). HP1γ is localized to euchromatin or to both euchromatin and heterochromatin, whilst HP1α and β are unambiguously localized to dense pericentromeric heterochromatin (12,13). Mice in which different HP1 isoform genes are knocked out show distinct phenotypes, indicating different functions of the isoforms. The HP1α-null mouse shows no phenotype (14), while knockout of HP1β is associated with genome instability and shows defects in neuronal development (15). Mice expressing low HP1γ levels show severe defects in spermatogenesis (16). The number of progenitor germ cells before meiosis are drastically reduced in the HP1γ-null mouse (17,18). HP1γ-null mice of the C57BL/6 background exhibit neonatal lethality (18). Knockdown of HP1γ, but not HP1α or HP1β, specifically leads to mitotic defects in human cultured cells (19). These studies strongly suggest that the functions of HP1 isoforms are distinct.

The CD of all the HP1 isoforms, as well as the full-length proteins, show similar affinity towards a histone H3 tail peptide (residues 1–15) containing K9me3 *in vitro* (20). Isoform-specific interacting factors as well as post-translational modifications have been reported to explain their distinct properties in light of their similar binding affinities (21–24). Recently, we have found that full-length HP1α, but not the CD alone, can bind to H3K9me3 in reconstituted nucleosomes with the aid of the CSD and HR (8). This characteristic property of HP1α for the recognition of H3K9me3 in the context of a nucleosome provides a clue to understand the isoform-specific functions.

In the present study, we have characterized the binding properties of HP1γ for H3K9me3 in nucleosomes, and found novel recognition properties of HP1γ towards H3K9me3. In contrast to HP1α, HP1γ could not recognize H3K9me3 in extended nucleosomes. On the other hand, under the conditions that cause nucleosomes to adopt a condensed state, HP1γ selectively recognized H3K9me3 even in nucleosomes. For the binding of HP1γ to condensed nucleosomes, the dimerization via CSD was found to be a prerequisite. We propose that this unique recognition of H3K9me3 in chromatin structure by HP1γ plays a key role in its specific function *in vivo*.

## MATERIALS AND METHODS

### Construction of expression plasmids

The cDNA of human HP1γ and HP1α was kindly provided by Dr Tokuko Haraguchi at the National Institute of Information and Communication Technology (Kobe, Japan). The cDNAs encoding full-length, truncated, and site-directed mutagenized HP1γ were subcloned into expression vector pGEX-6P1 in frame. The constructs for the full-length histones were described elsewhere (25). All the cDNAs, unless otherwise stated, were amplified by PCR via ordinary methods. The DNA sequences of all the plasmids constructed or used in the present study were confirmed by dideoxy sequencing (26).

### Purification of proteins

Recombinant histones and H3K9me3 were prepared as described elsewhere (8,25). Full-length, truncated and mutagenized GST-HP1γ and HP1α were expressed and purified as described by Mishima *et al*. (8). The GST-tag at the N-terminus of GST-HP1γ and α was removed as described (8). Full-length H1.2, H1.3, H1.4 and H1.5 were expressed without tag, and purified as described elsewhere (27).

### Preparation of native nucleosomes

Native oligo-nucleosomes were prepared as described (27) with slight modifications. HeLa cells (1 × 10^8^ cells) were suspended with 4 ml of buffer A comprising 0.5 mM EDTA, 2 mM MgCl_2_, 0.1% (v/v) protease inhibitor (PI) (Nakalai Tesque, Japan), 2 mM 2-mercaptoethanol, 10 mM Tris–HCl, pH 7.8, and centrifuged at 1500 × g for 2 min at 4°C. The pellet was suspended with 4 ml of buffer A containing 0.025% (w/v) Triton-X100. The suspension was centrifuged at 1500 × g for 2 min at 4°C, and then the pellet was washed twice with 4 ml of buffer A. The precipitate was re-suspended with 0.75 ml of buffer comprising 1 mM KCl, 1 mM CaCl_2_, 0.1% (v/v) PI, 0.34 M sucrose, 10 mM Tris–HCl, pH7.4, and then treated with 4.3 U/ml of micrococcal nuclease at 37°C for 15 min to prepare oligo-nucleosomes. The reaction was terminated by adding final concentration of 5 mM EDTA and then the mixture was centrifuged at 2000 × g for 2 min at 4°C. The precipitate was then suspended with 0.7 ml buffer comprising 1 mM EDTA, 0.1% (v/v) PI, 10 mM Tris–HCl, pH 7.4, and then centrifuged at 2000 × g for 2 min at 4°C. The supernatant fraction was recovered as oligo-nucleosomes. Oligo-nucleosomes thus prepared were dialyzed against 0.1% (v/v) PI and 10 mM Tris–HCl, pH 7.4, at 4°C. The concentrations of native nucleosomes were expressed as DNA concentrations calculated from the absorbance at 260 nm.

### Nucleosome reconstitution

Histone octamers were reconstituted as described elsewhere (25,28). Nucleosomes were reconstituted with histone octamers and DNA by a salt-dialysis method (28). DNA sequences used for reconstituting tetra-nucleosomes and mono-nucleosomes, of which length are 694 and 193 bp, respectively, are described elsewhere (8). The reconstituted nucleosomes were purified by glycerol density gradient centrifugation as described elsewhere (25). The concentrations of nucleosomes were expressed as DNA concentrations calculated from the absorbance at 260 nm.

### Pull down assay determining nucleosome-binding activity of HP1

Pull down assay was performed as described (8). In a standard binding assay mixture, 4 pmol (amount converted to that of a nucleosome particle with a histone octamer) of reconstituted nucleosomes or 1.2 μg of native nucleosomes prepared from HeLa cells, unless otherwise indicated, were incubated with 80 pmol of GST-HP1γ-bound glutathione (GSH) Sepharose (GE Healthcare) of 10 μl packed volume, in 20 μl of a binding buffer comprising 50 mM NaCl, 0.2 mM DTT, 0.2 mM phenylmethylsulfonyl fluoride, 0.1% (w/v), Nonidet P-40, 20% (v/v) glycerol, and 20 mM Tris–HCl, pH 7.4, with or without 1 mM EDTA. Reaction mixtures were incubated for 30 min at room temperature. The input, unbound, wash and bound fractions were electrophoresed in an 18% SDS-polyacrylamide gels, and then the protein bands were stained with Lumitein (Biotium, CA, USA) and determined in a fluoro-imager, FLA9500 (GE Healthcare, Japan). The density of bands corresponding to core histones was quantitated by Image Gauge V4.0 software (GE Healthcare).

### Sucrose density gradient centrifugation

Sucrose density gradient centrifugation was performed as described elsewhere (8). In brief, 75 μl of reaction mixtures containing 0.3 nmol of HP1γ or HP1α and 24 pmol (amount converted to that of a nucleosome particle with a histone octamer) un-methylated or H3K9me3 tetra-nucleosomes in the binding buffer were subjected to 15–40% (w/v) sucrose density gradient. After the centrifugation with a RPS50-2 rotor at 30 000× rpm for 14 h at 4°C, fractions were collected from the bottom of the tubes. The proteins in the fractions were electrophoresed in 18% SDS-polyacrylamide gels, and the protein bands were stained with Lumitein.

### Western blotting

Western blotting analyses were performed as described elsewhere (29). In brief, after proteins were separated in a SDS-polyacrylamide gel, the proteins were electrophoretically transferred to a nitrocellulose membrane (Pall, Japan), and then incubated with specific antibodies, as indicated. The antibodies that bound to specific antigens were detected with alkaline phosphatase-conjugated secondary antibodies and a color reaction buffer containing 50 μg/ml indolyl phosphate and 1 mg/ml *p*-nitro blue tetrazolium chloride. Each band was quantitated with Quantity One software (Bio-Rad, Japan).

### Gel shift assays

H1 isoforms (10, 20, 40 or 80 nM) were mixed with tetra-nucleosomes (20 nM of histone octamer) in the binding buffer with 1 mM EDTA. After the incubation, the mixtures were electrophoresed in a 0.7% agarose gel with 0.5× TBE, and then DNA bands were stained with GelGreen (Biotium) and quantitated with a fluoro-imager, FLA9500, as described by Mishima *et al*. (8).

### Antibodies

Anti-HP1γ antibodies from Bioacademia, Japan, code # 70-225 for western blotting was used, and from Active Motif, Japan, clone 2MOD-1G6, code # 39981 for indirect immunostaining. Anti-H3K9me3 (Active motif, code # 39161), anti-H1.2 (Abcam, Japan, code # ab17677), anti-H1.5 (Abcam, code # ab24175), and anti-H4 (Abcam, code # ab10158) antibodies were used for detection of each protein.

### Indirect immunostaining

HeLa cells were cultured in Dulbecco's modified Eagle's medium (DMEM), supplemented with 10% (v/v) fetal bovine serum (FBS) on gelatin-coated cover glasses #1S (Matsunami Co. Ltd, Japan). Upon 70% confluency, cells were briefly washed with 1x phosphate buffered saline (PBS) and then fixed with 4% (v/v) paraformaldehyde in DPBS at room temperature for 20 min. The fixed cells were permeabilized with 0.5% (w/v) Triton X-100 at room temperature for 20 min, washed with 1x PBS with 0.5% (w/v) Tween-20 (PBST), and then incubated with primary antibodies in PBS with 2% (w/v) bovine serum albumin (BSA) at 4°C overnight. Slides were washed three times with 1x PBST, and then were incubated with 1:500 dilution of DyLight 488 conjugated donkey anti-mouse antibodies for detecting HP1γ and DyLight 594 conjugated donkey anti-rabbit antibodies (Pierce, Japan) for detecting H1.2 and H1.5 in PBS-T with 2% (w/v) BSA at room temperature for 1 h. After incubation, slides were washed three times with 1x PBST, and then were counterstained with DAPI and mounted in 100% glycerol with 2% (w/v) *n*-propyl gallate added as an anti-fading agent. Microscope images were taken with a DeltaVision (Applied Precision/GE Healthcare) microscope at 0.2 μm section spacing, and deconvolved with the softWoRx suite. Chromatic aberration was corrected using cross-correlation of DAPI images in all three color channels. Images are shown with linear scaling only.

### Cross-correlation

Otsu thresholding of maximum-intensity projections of the DAPI channel from each nucleus was used to determine a foreground/background segmentation of the entire volume. Pixels corresponding to the DAPI channel foreground were selected from all channels for correlation analysis. Correlation coefficients were determined pairwise between all channels using the corr2 function from the GNU Octave ‘Image’ package. Scatter plots were constructed in GraphPad Prism.

### H1-dependent HP1γ precipitation accompanying tetra-nucleosomes

The indicated type of H1 (0.8 μM) was mixed with 0.2 μM tetra-nucleosomes (amount converted to that of a nucleosome particle with a histone octamer) in 10 μl of the binding buffer. To the mixture of tetra-nucleosomes and H1, full-length or truncated HP1γ (0.5 μM) were added. After centrifugation, the supernatant and precipitate fractions were separated in 18% polyacrylamide gels, and the gel was stained with Lumitein. The protein bands were visualized in a fluoro-imager, FLA9500.

### Gel filtration

Purified HP1 without GST tag (50 μg) was separated by size exclusion chromatography in Superdex 200 (1 × 30 cm, GE healthcare) equilibrated with 0.1 M NaCl, 10% (v/v) glycerol, 1 mM DTT, 0.2 mM EDTA, 20 mM HEPES-Na, pH 7.0, at constant flow rate (0.35 ml/min) as described elsewhere (30). Elution of the protein was monitored by absorbance at 280 nm.

## RESULTS

### Isoform-specific HP1 binding to extended nucleosomes

It was reported that HP1 isoforms play distinct functions *in vivo* (14,16–18). However, consistent with previous reports (20), we found the binding affinity of HP1α and HP1γ towards a histone H3 tail peptide with K9me3 determined by isothermal titration calorimetry (ITC) was in a similar range (Supplementary Figure S1). The discrepancy between *in vivo* functions and *in vitro* binding properties suggest that there exists mechanism(s) other than simple recognition by the CD that regulates an isoform-specific localization and function *in vivo*.

**Figure 1.**
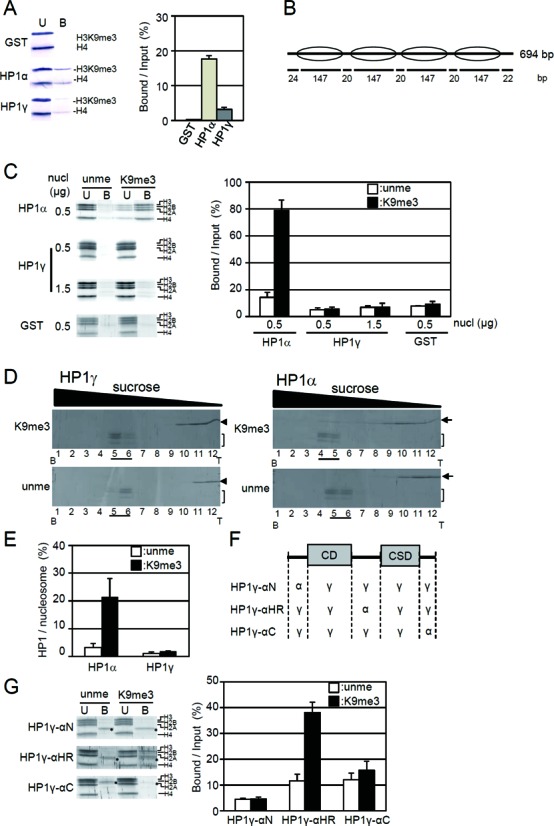
Distinct binding of HP1γ and HP1α to extended nucleosomes. (**A**) HP1γ binding to native oligo-nucleosomes prepared from HeLa cells. GSH-Sepharose bound with GST-HP1γ (HP1γ), GST-HP1α (HP1α), or GST (GST) was mixed with native oligo-nucleosomes and then pulled down. The unbound (U) and bound fractions (B) were subjected to SDS-PAGE, and H3K9me3 and histone H4 in the fractions were detected with anti-H3K9me3 (H3K9me3) and anti-H4 antibodies (H4) (left panel). Western blot data is taken from the whole gel image shown in Supplementary Figure S13. The amounts of H3K9me3 in the bound fractions over input (%) are shown as mean ± S. E. (*n* = 3) (right panel). (**B**) Schematic illustration of reconstituted tetra-nucleosomes. The position of the nucleosomes on DNA is indicated by ellipses. Length (bp) of linker and nucleosome core region DNA are shown below. (**C**) Binding of GST-HP1 to the reconstituted tetra-nucleosomes. Indicated amounts of unmethylated or H3K9me3 tetra-nucleosomes (nucl) were incubated with GST-HP1γ (HP1γ) or GST-HP1α (HP1α) bound to GSH-Sepharose. Unbound (U) and bound nucleosomes (B) were separated in an 18% SDS polyacrylamide gel, stained (left panel), and positions of core histones are indicated. The amounts of core histones were quantitated, and the relative amount of core histones with unmethylated H3 (open columns) or H3K9me3 (closed columns) in the bound fraction over input (%) were calculated and shown as mean ± S.E. (*n* = 3) (right panel). The whole gel for the pull-down assay is shown in Supplementary Figure S13. (**D**) The interaction of HP1γ (left panel) or HP1α (right panel) with unmodified H3 (unme) or H3K9me3 tetra-nucleosomes (K9me3) was analyzed by sucrose density gradient (15–40% (w/v) sucrose). Fractions were collected from the bottom of the tubes (B) and the proteins were separated in 18% polyacrylamide gels. HP1γ (arrow heads), HP1α (arrows), and core histones (brackets) were stained with Lumitein. (**E**) The amounts of HP1γ and HP1α in nucleosome fractions (underlined) were densitometrically determined from three independent centrifugation experiments, and the ratios to those of core histones in nucleosome fractions were calculated and are shown. The values are mean ± S.E. (*n* = 3). (**F**) Schematic illustration of the chimeric HP1 proteins used in the present study. (**G**) Nucleosome binding of chimeric HP1. The binding activity was analyzed as in panel (C). The core histones and the gels were shown in the left panel, and the core histones in bound fraction over input are shown as mean ± S.E. (*n* = 3) in the right panel. Asterisks indicate the position of degraded products of GST-HP1.

In *Xenopus*, it was reported that HP1α but not HP1γ can bind to chromatin prepared from chicken erythrocytes that may carry heterogeneous modifications on core histones (31). To determine whether or not this HP1 isoform-specific binding is observed also in mammals, we purified human recombinant HP1α and HP1γ (Supplementary Figure S2) (8). The binding of the crude oligo-nucleosomes prepared from HeLa cell nuclei to GST-HP1α or GST-HP1γ anchored on GSH-Sepharose was determined in the absence of MgCl_2_, which is known to induce compaction of nucleosomes (32,33). GST-HP1α-coupled Sepharose trapped about 18% of input H3K9me3-nucleosomes, while GST-HP1γ-coupled Sepharose trapped a very low level of the nucleosomes (Figure 1A). This result shows HP1γ hardly binds to H3K9me3 in extended nucleosomes, while HP1α does.

Nucleosomes prepared from cultured cells carry not only additional proteins but also modifications other than H3K9me3 (34). Phosphorylation of Ser 10 of H3 inhibits the interaction between HP1 and the N-terminal tail peptide of histone H3 (20). To simplify the system, we reconstituted tetra-nucleosomes with the 601.2 × 4 DNA sequence and recombinant histones H2A, H2B, H4 and H3 or H3K9me3 (Figure 1B) (8), and the binding activity of HP1 to the nucleosomes in an extended form was determined. Tetra-nucleosomes reconstituted with H3K9me3 (H3K9me3 tetra-nucleosomes) selectively bound to GST-HP1α, as reported previously (8). On the other hand, H3K9me3 tetra-nucleosomes could not significantly bind to GST-HP1γ on beads (Figure 1C), which was similar to the results with oligo-nucleosomes prepared from HeLa cells (Figure 1A). The amount of tetra-nucleosomes bound to GST-HP1γ on beads was similar to that binding to GST beads alone (Figure 1C). Even when a three-fold higher amount of H3K9me3 tetra-nucleosomes was used, the amount of H3K9me3 tetra-nucleosomes that bound to HP1γ did not increase (Figure 1C). These results indicate that HP1γ cannot bind to H3K9me3 in a nucleosome context.

As it was reported that GST is likely to form dimers (35), it may be possible that this dimerization affected the binding properties of HP1 isoforms to the H3K9me3 tetra-nucleosomes. To eliminate the GST-tag dimerization effect on the binding properties of HP1 toward H3K9me3 tetra-nucleosomes, HP1α and HP1γ with GST-tags removed (Supplementary Figure S2) were incubated with H3K9me3 tetra-nucleosomes, and then the reaction mixtures were separated by sucrose density gradient centrifugation. As shown, HP1α significantly bound to H3K9me3 tetra-nucleosomes, as described by Mishima *et al*. (8); however, HP1γ did not co-migrate with H3K9me3 tetra-nucleosomes (Figure 1D and E). GST-tags did not affect the binding properties of HP1α or HP1γ, and thus HP1 showed isoform-specific binding activity when either native or reconstituted nucleosomes were used as a substrate.

Since the primary sequences of the CD and CSD of HP1 isoforms are highly conserved (Supplementary Figure S2), the non-conserved parts of HP1α and HP1γ may determine the isoform-specific binding properties. As the N-termini, HR, and C-termini of HP1 isoforms are divergent (Supplementary Figure S2), we prepared three chimeric HP1γ proteins, in which non-homologous regions were replaced either with the N-terminus (HP1γ-αN), HR (HP1γ-αHR), or C-terminus (HP1γ-αC) of HP1α, respectively (Figure 1F). Among the chimeric recombinants, only the recombinant HP1γ-αHR, in which HP1γ (76–109) was replaced with HP1α (76–119), selectively bound to H3K9me3 tetra-nucleosomes (Figure 1G), however, the binding level was not fully recovered to that of HP1α (compare panels C and E in Figure 1). The result suggests that the HR is one of the determinants for the isoform-specific binding activity. It was reported that the HR of HP1α contributes to its DNA-binding activity, and this DNA-binding activity is necessary for the selective recognition of H3K9me3 tetra-nucleosomes by HP1α (8). On the contrary, HP1γ possesses little or no DNA-binding activity (Supplementary Figure S3), as Nishibuchi *et al*. previously reported (24). This lack of DNA-binding activity of HP1γ may be one of the reasons that HP1γ could not bind to H3K9me3 tetra-nucleosomes. Interestingly, when the CSD was deleted from HP1γ, the DNA-binding and selective binding towards H3K9me3 tetra-nucleosomes were observed, while CD of HP1γ by itself showed neither of these binding activities (Supplementary Figure S4B and C). The results indicate that the CSD is negatively regulating the DNA-binding activity of the HR of HP1γ. Thus, similar to HP1α (8), the binding activity of HP1γ towards nucleosomes is due to the balance between the DNA-binding by the hinge region, the repressive activity of the CSD, and the H3K9me3-binding activity via the CD.

### HP1γ is able to bind to H3K9me3 in nucleosomes in the presence of Mg^2+^ ion

**Figure 2.**
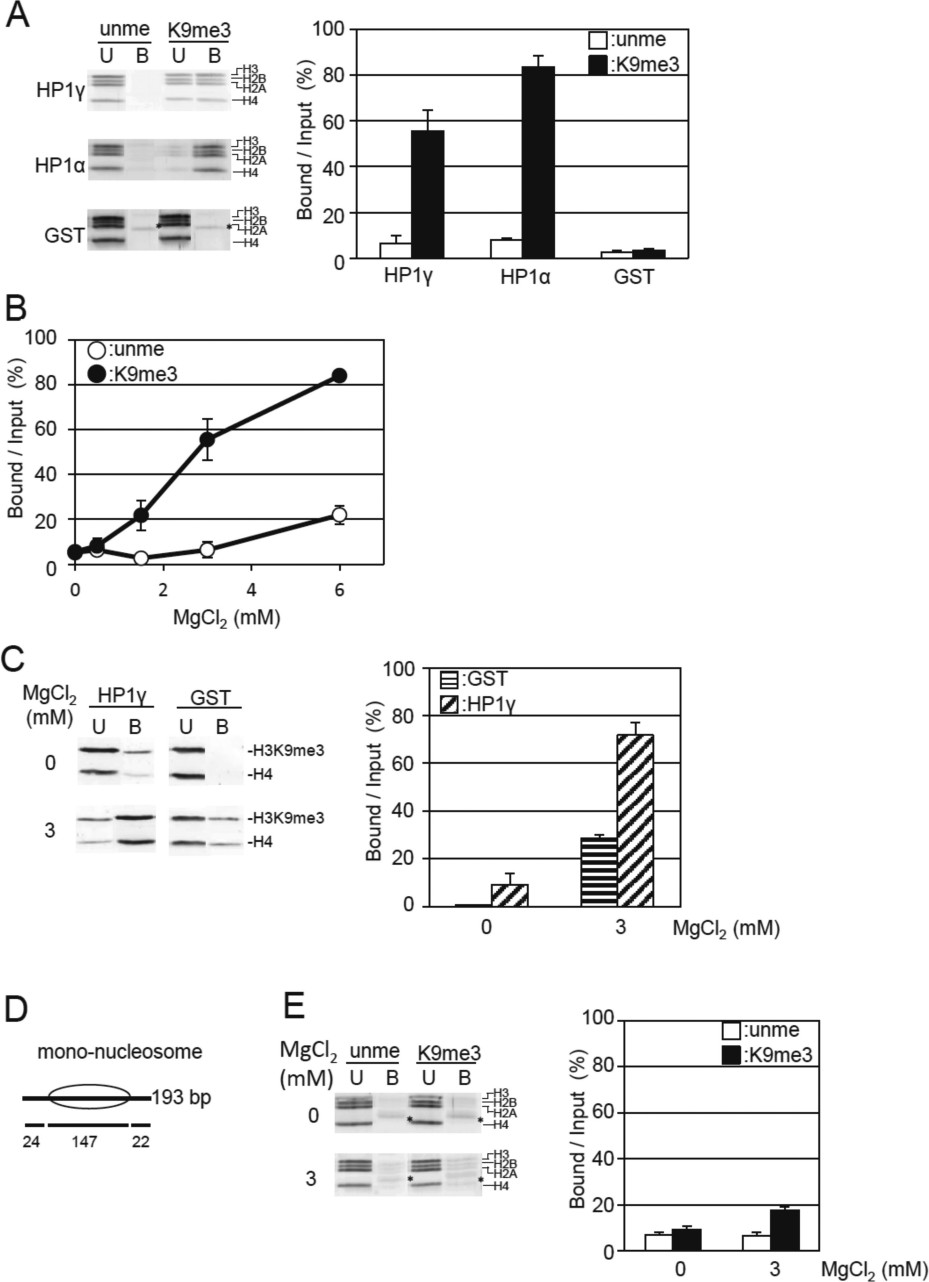
Magnesium ion induces the selective binding of HP1γ to H3K9me tetra-nucleosomes. (**A**) Effect of MgCl_2_ on the binding of HP1γ to H3K9me3 tetra-nucleosomes. Unmethylated (unme) or H3K9me3 tetra-nucleosomes (K9me3) were incubated with GST-HP1γ (HP1γ) or GST-HP1α (HP1α) bound to GSH-Sepharose in the presence of 3 mM MgCl_2_, 50 mM NaCl. Unbound (U) and bound nucleosomes (B) are visualized (left panel), and the nucleosome core histones indicated by brackets were quantitated as in Figure 1C. Relative amounts of core histones in the bound fraction per input (%) were calculated and shown as mean ± S.E. (*n* = 3) (right panel). (**B**) MgCl_2_ concentration-dependent selective binding of HP1γ to H3K9me3 tetra-nucleosomes. The binding activity of HP1γ to unmethylated H3 (open circles) or H3K9me3 tetra-nucleosomes (closed circles) was determined and shown as mean ± S.E. (*n* = 3) under the indicated concentration of MgCl_2_ in the presence of 50 mM NaCl by pull-down assay as described in Figure 1C. (**C**) MgCl_2_ concentration-dependent binding of HP1γ to native oligo-nucleosomes with H3K9me3 modification. GST-HP1γ (HP1γ) or GST (GST) bound to GSH-Sepharose was mixed with native oligo-nucleosomes and then pulled down under the condition including 50 mM NaCl, in the absence or presence of 3 mM MgCl_2_. The H3K9me3 and histone H4 in the unbound (U) and bound fractions (B) were analyzed as in Figure 1A (left panel). The amounts of H3K9me3 in the HP1γ-bound fractions over the input (%) are shown as mean ± S.E. (*n* = 3) (right panel). (**D**) Schematic illustration of reconstituted mono-nucleosomes. The position of the nucleosomes on DNA is indicated by ellipses. Length (bp) of linker and nucleosome core region DNA are shown below. (**E**) Effect of MgCl_2_ on the binding of HP1γ to H3K9me3 mono-nucleosomes. The binding of ummethylated H3 and H3K9me3 mono-nucleosomes (4 pmol of nucleosome core particle) to GST-HP1γ (80 pmol) bound to GSH-Sepharose in 10 μl of the reaction mixture was examined as in Figure 1C, with 50 mM NaCl, in the absence or presence of 3 mM MgCl_2_ (left panel). The binding activity of HP1γ to tetra-nucleosomes reconstituted with unmethylated H3 (open circles) or H3K9me3 (closed circles) was determined and shown as mean ± S.E. (*n* = 3) under the indicated concentration of MgCl_2_ by pull-down assay as described in Figure 1C.

HP1γ did not bind to extended nucleosomes containing H3K9me3 (Figure 1). In contrast, it has been reported that HP1γ co-precipitates with nucleosomes containing H3K9me3 (36), and the localization of HP1γ at the specific gene locus of *D4Z4* depends on the H3K9 methyltransferase Suv39 (37). These reports, nevertheless, suggest the possibility that the localization of HP1γ in an H3K9me3-dependent manner is due to its selective recognition of H3K9me3. In addition to the difference in DNA-binding ability of HP1α and HP1γ (Supplementary Figure S3), the length of the HR of HP1γ is shorter than that of HP1α (Supplementary Figure S2A). For this, we assumed that the spatial distance between the CD and CSD in HP1γ may be one of the limiting factors for the selective binding. Thus, we examined whether or not the condensation of nucleosomes, which changes the distance between the H3K9me3 in adjacent cores of the nucleosome, is regulating the HP1γ recognition of H3K9me3. As divalent cations such as Mg^2+^ induces nucleosome compaction (32,33), we determined the interaction between HP1γ and nucleosomes in the presence of MgCl_2_ (Figure 2).

We examined the effect of MgCl_2_ on the nucleosome precipitation by high-speed centrifugation at 10 000 × g. Above 3 mM MgCl_2_, ∼80% of the tetra-nucleosomes were precipitated irrespective of H3K9 methylation status (Supplementary Figure S5A), indicating that 3 mM MgCl_2_ is sufficient to condense tetra-nucleosomes. In the presence of 3 mM MgCl_2_, GST-HP1γ-Sepharose selectively precipitated H3K9me3 tetra-nucleosomes (Figure 2A). The H3K9me3-selective binding was observed above 1.5 mM MgCl_2_ (Figure 2B), under which conditions about 20% of the nucleosomes were precipitated by high-speed centrifugation (Supplementary Figure S5A). The elution position of HP1γ from a gel filtration column was not affected by the presence of 3 mM MgCl_2_ (Supplementary Figure S6), indicating that the MgCl_2_-dependent selective binding to H3K9me3 tetra-nucleosomes was not due to the multimerization state of HP1γ. As DNA-binding activity of HP1γ was not observed even in the presence of 3 mM MgCl_2_ (Supplementary Figure S3B), as well as in the absence of MgCl_2_ (Supplementary Figure S3A), the effect of MgCl_2_ on the nucleosome binding is, at least, not depend on the DNA-binding activity. Furthermore, the presence of a GST-tag at the N-terminus did not affect the selective binding of HP1γ to H3K9me3 tetra-nucleosomes in the presence of MgCl_2_, as HP1γ whose GST-tag was removed also selectively precipitated H3K9me3 tetra-nucleosomes (Supplementary Figure S7). The addition of MgCl_2_ also enhanced the binding activity of HP1γ to oligo-nucleosomes prepared from HeLa nuclei (Figure 2C). Taken together, these results indicate that HP1γ selectively binds to H3K9me3 in a nucleosomal context in a nucleosome condensation-dependent manner.

GST-HP1α binding to H3K9me3 tetra-nucleosomes was not significantly affected by the addition of 3 mM MgCl_2_, while that to unmodified ones was reduced (compare Figures 1C and 2A). This indicates that MgCl_2_ increased the binding specificity of HP1α. However, when the amount of tetra-nucleosomes or HP1α was reduced to be a half in the experiments, the binding activities to both the unmodified and H3K9me3 tetra-nucleosomes were not significantly changed (Supplementary Figure S8). Thus, under the conditions examined, the drastic change in the binging activity by adding MgCl_2_ is specifically observed in HP1γ.

To evaluate whether the effect of Mg^2+^ ion on the binding was dependent on intra- or inter-nucleosome effects, we prepared unmethylated H3 and H3K9me3 mono-nucleosomes (Figure 2D), and then determined the binding to GST-HP1γ. Above 2 mM MgCl_2_, 40–50% of the input mono-nucleosomes were precipitated by high-speed centrifugation (Supplementary Figure S5B). Although the amount of H3K9me3 mono-nucleosomes was significantly elevated compared to the GST-HP1γ-bound fraction in the presence of 3 mM MgCl_2_ (Figure 2E), the amount was much lower that observed with tetra-nucleosomes (Figure 2A). Since mono-nucleosomes can only form inter-nucleosome aggregations, these observations indicate that the condensation of tetra-nucleosomes due to intra-nucleosome effects is the major cause for the acquisition of the selective binding of HP1γ to the H3K9me3 in nucleosomes.

### Linker histone H1 facilitates the binding of HP1γ to H3K9me3 in nucleosomes

**Figure 3.**
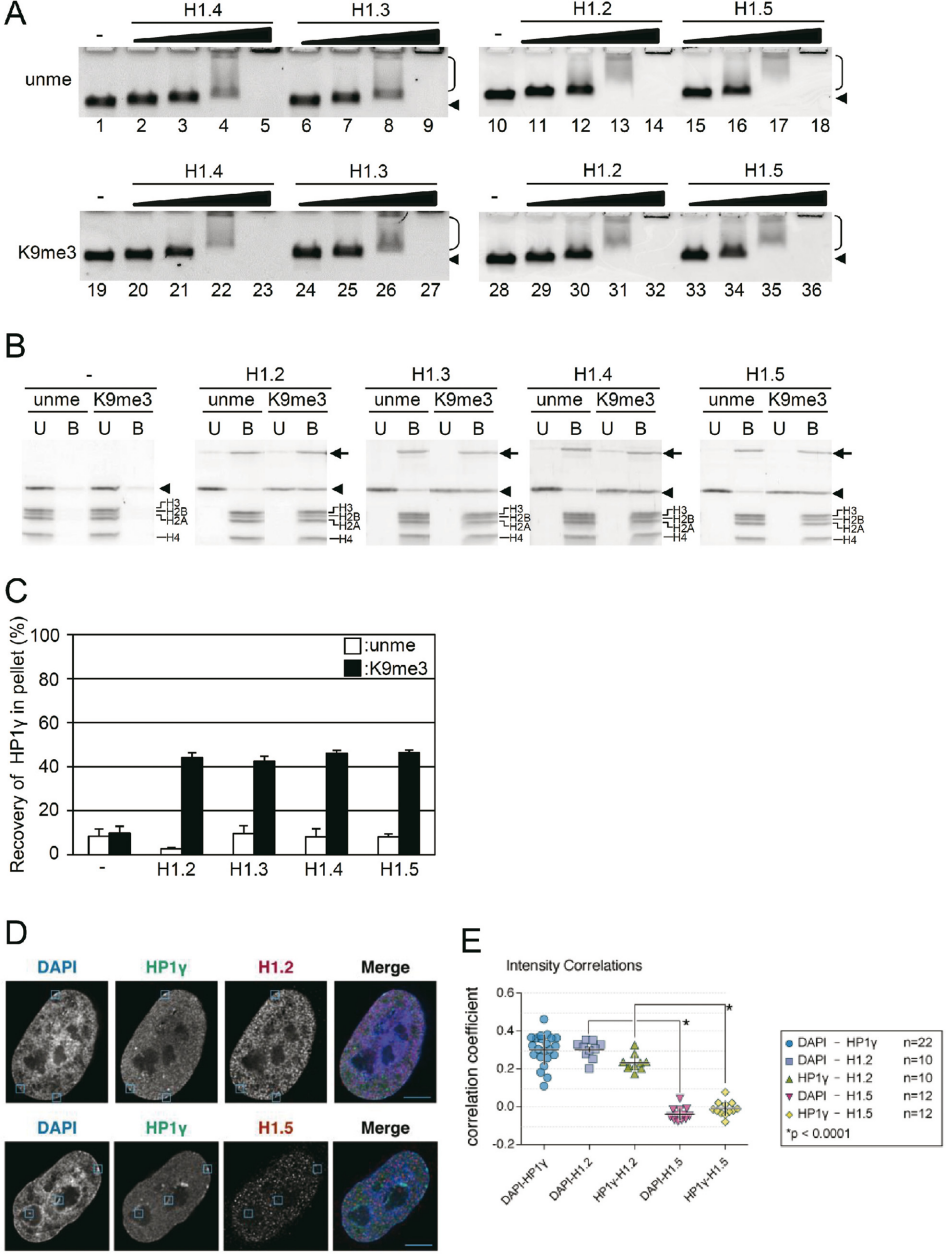
H1 isoforms and the nucleosome binding of HP1γ. (**A**) Tetra-nucleosome binding activity of H1 isoforms. Tetra-nucleosomes were incubated without H1 (lanes 1, 10, 19 and 28), with H1.2 (lanes 11–14, and 29–32), H1.3 (lanes 6–9, and 24–27), H1.4 (lanes 2–5, and 20–23), or H1.5 (lanes 15–18, and 33–36). To unmethylated H3 (unme; upper panel) or H3K9me3 (K9me3; lower panel) tetra-nucleosomes, molar ratio of 1/2 (lanes 2, 6, 11, 15, 20, 24, 29 and 33), 1/1 (lanes 3, 7, 12, 16, 21, 25, 30 and 34), 2/1 (lanes 4, 8, 13, 17, 22, 26, 31 and 35), or 4/1 (lanes 5, 9, 14, 18, 23, 27, 32 and 36) of H1 isoforms to nucleosome core particles was added, incubated, and then electrophoresed in 0.7% agarose gels. Arrowheads and brackets indicate the positions of free tetra-nucleosomes and shifted nucleosomes, respectively. (**B**) H1 induced selective binding of HP1γ to H3K9me3 tetra-nucleosomes. HP1γ and unmethylated (unme) or H3K9me3 tetra-nucleosomes (K9me3) were incubated in the absence or presence of a fourfold molar excess of H1. Unbound (U) and bound (B) tetra-nucleosome fractions were separated by centrifugation, SDS-PAGE, and then the amount of HP1γ in each fraction was densitometrically determined. The arrows, arrowheads, and brackets indicate H1, HP1γ, and histones, respectively. (**C**) Relative amounts of HP1γ in the bound fractions were calculated, and mean ± S.E. (*n* = 3) are shown. (**D**) Co-localization of HP1γ and H1 isoforms. Immunofluorescence staining of DAPI, HP1γ and H1.2 (upper row) or H1.5 (lower row) were merged. A single Z section from deconvolved 3D stacks is shown. Inset boxes indicate same areas in all three channels to facilitate intensity comparisons. Scale bar indicates 5 μm. (**E**) Scatter plot of correlation coefficients between channels. DAPI and HP1γ were compared in 22 nuclei, among which 10 were stained for H1.2 and 12 for H1.5. Bars in scatterplots indicate the mean ± S.D.

Linker histone H1 (H1) is known to be a key molecule for chromatin condensation *in vivo* (38–41). We prepared four different isoforms of recombinant H1 (Supplementary Figure S2) and determined their effect on the binding activity of HP1γ. All the prepared H1 isoforms shifted the migration of unmethylated H3 and H3K9me3 tetra-nucleosomes in gel shift assay, when H1 was present in 4-fold molar excess compared to nucleosome core particles (Figure 3A). In the presence of 4-fold molar excess of H1, more than 95% of reconstituted tetra-nucleosomes were precipitated by high speed centrifugation, irrespective of H1 isoform and H3K9 methylation status (Supplementary Figure S9). Under identical conditions, neither HP1γ (with GST-tag removed), nor H1, nor a mixture of the two were precipitated, but were all recovered in the supernatant fraction (Supplementary Figure S10). Therefore, the addition of H1 only affected the condensation of tetra-nucleosomes, and did not alter the solubility of HP1γ. This allows us to conclude that H1 modulation of the interaction between HP1γ and nucleosomes can be examined by centrifugation.

In the presence of H1, HP1γ was selectively co-precipitated with H3K9me3 tetra-nucleosomes (Figure 3B), and the amount of HP1γ that co-precipitated with H3K9me3 nucleosomes was similar among all the H1 isoforms examined (Figure 3C). A pull-down assay with GST-HP1γ-Sepharose also showed selective binding of H3K9me3 tetra-nucleosomes in the presence of any of the H1 isoforms (Supplementary Figure S11). These results indicate that nucleosome condensation by H1 allows the selective binding of HP1γ, and that the effect was isoform-independent.

To analyze the region of H1 responsible for the selective binding of HP1γ to H3K9me3 tetra-nucleosomes, truncated histone H1.3 was prepared. As it has been reported that the C-terminal region of histone H1 is responsible for chromatin binding and stabilization of its histone fold (38,40,41), we prepared two types of truncation lacking the N-terminal 36 amino acid residues (ΔN) and the C-terminal 110 amino acid residues (ΔC) (Supplementary Figure S2 and S12A). As expected, ΔC could not shift the band of tetra-nucleosomes by gel shift assay irrespective of H3K9 methylation, while ΔN could (Supplementary Figure S12B), indicating that the C-terminal region of H1 is required for the compaction of nucleosomes. Despite this, ΔN was still able to enhance the selective binding of HP1γ to H3K9me3 tetra-nucleosomes (Supplementary Figure S12C). Taken together, our data shows that HP1γ selectively binds to H3K9me3 tetra-nucleosomes condensed by the C-terminal region of H1.

Next, we examined the co-localization of H1 and HP1γ *in situ*, by immunofluorescence staining of fixed cells. As shown previously (13), immunostaining with specific antibodies showed that HP1γ partially but co-localized with DAPI-dense regions with statisticaly signifincane (Figure 3D). H1.2 is reported to be enriched at major satellite sequences (42), specifically enriched at the X chromosome (43), and its abundance at distal promoter regions is inversely proportional to expression level (44). We found that H1.2, whose localization was highly correlated with DAPI-dense regions, was co-localized with HP1γ (Figure 3D and E). This is consistent with a previous report describing that global distribution of H1.2 is similar to that of H3K9me3 (42). On the contrary, H1.5 was co-localized neither with HP1γ (Figure 3D and E) nor with DAPI. In contrast to *in vitro* analysis, in which all the isoforms of H1 examined allowed HP1γ to bind to nucleosomes in H3K9me3-dependent manner, HP1γ showed preferential colocalization with a specific isoform of H1 in nuclei of cultured cells. As shown in the present study, HP1γ did not induce aggregation of nucleosomes by binding to H3K9me3, but rather HP1γ binding to H3K9me3 tetra-nucleosomes depends on their condensation state. By an unknown mechanism, H1.5 may be excluded from the DAPI-dense heterochromatin in nuclei, and thus HP1γ was not able to be recruited to the sites. Based on these results, we propose that HP1γ is not a heterochromatin inducer but is recruited to the preformed heterochromatin.

### Dimerization of HP1γ through CSD is necessary for binding to condensed H3K9me3 tetra-nucleosomes

**Figure 4.**
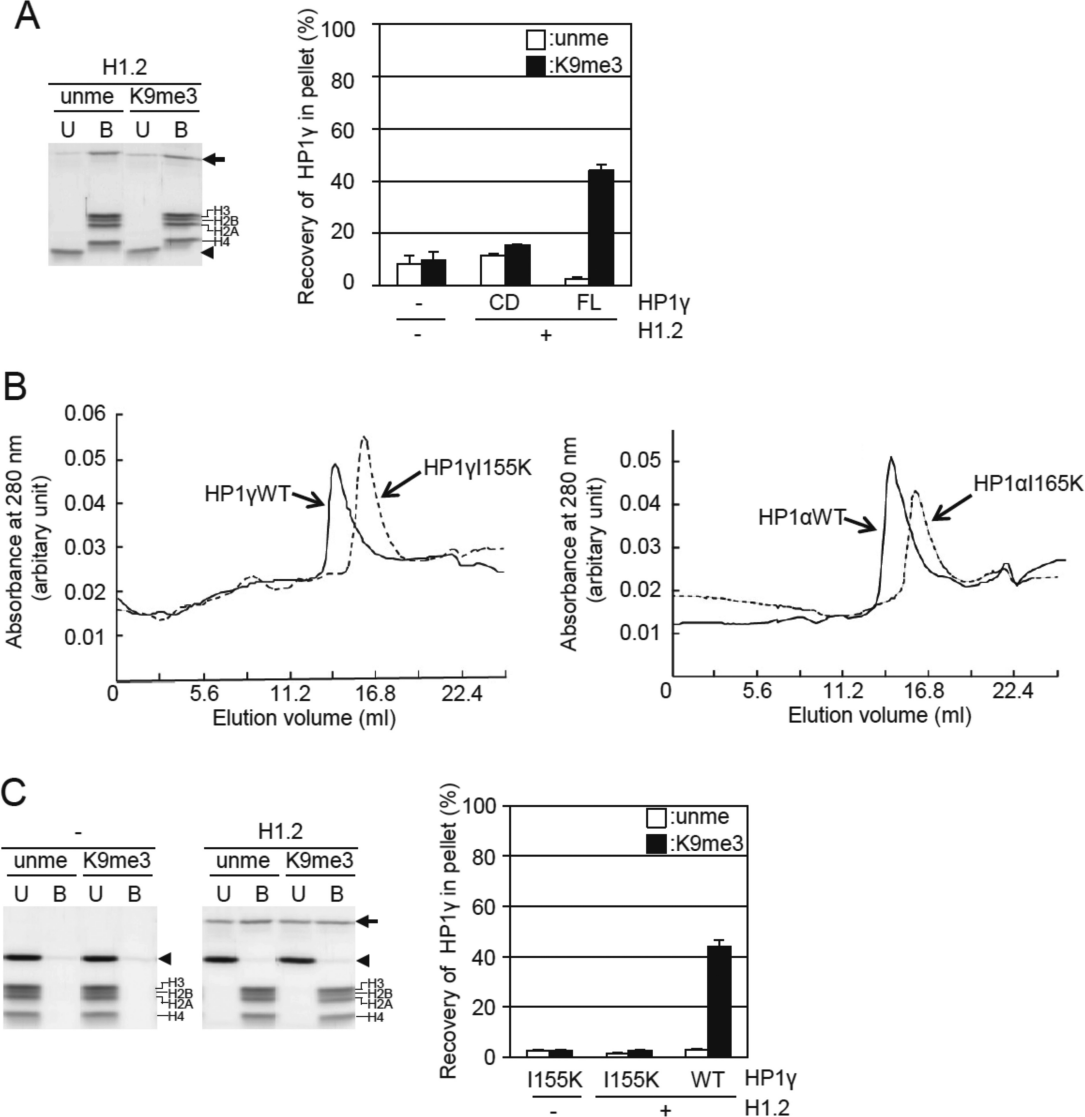
Effect of dimerization of HP1γ through the CSD on the selective binding of H3K9me3 tetra-nucleosomes. (**A**) Binding of the CD of HP1γ to tetra-nucleosomes in the presence of H1.2. The binding was analyzed as in Figure 3B. The CD of HP1γ and unmethylated (unme) or H3K9me3 tetra-nucleosomes (H3K9me3) were incubated in the absence or presence of 4-fold molar excess of H1. Unbound (U) and bound (B) tetra-nucleosome fractions were separated by centrifugation, electrophoresed, and then the amount of HP1γ in each fraction was densitometrically determined. The arrows, arrowheads, and brackets indicate H1, the CD of HP1γ and H1.2, respectively (left panel). Relative amounts of the CD of HP1γ in the bound fractions were calculated, and means ± S.E. (*n* = 3) are shown. The values of the binding activity of HP1γ (FL) to tetra-nucleosomes are taken from Figure 3C. (**B**) Effect of the mutation in the CSD on apparent molecular sizes of the HP1α and HP1γ. Gel filtration of HP1γ (solid line) and HP1γ I155K (dotted line) (left panel) and HP1α (solid line), and HP1α I165K (dotted line) (right panel). (**C**) Binding of HP1γ (I155K) to tetra-nucleosomes in the presence of H1.2. Unbound (U) and bound (B) tetra-nucleosome fractions were separated by centrifugation, SDS-PAGE, and then the amount of HP1γ in each fraction was densitometrically determined. The arrows, arrowheads, and brackets indicate H1, HP1γ with I155K and H1.2, respectively (left panel). Relative amounts of HP1γ with I155K in the bound fractions were calculated, and means ± S.E. (*n* = 3) are shown. The values of the binding activity of HP1γ (FL) to tetra-nucleosomes are taken from Figure 3C.

Given that HP1γ recognizes H3K9me3 in condensed but not extended nucleosome structures, we expected that a physical closer spatial positioning of two CD in HP1γ dimer, compared with HP1α dimer, could be the reason for the selective binding of HP1γ to H3K9me3. As it has been reported that the CD by itself, in addition to CSD, is responsible for the dimerization of HP1 (9,10), we examined whether HP1γ (1–75) can selectively recognize the condensed H3K9me3 tetra-nucleosomes or not. As shown, HP1γ (1–75) could not bind to H3K9me3 tetra-nucleosomes condensed by H1.2 (Figure 4A). This indicates that a region other than the CD of HP1γ is necessary for the selective binding to H3K9me3 in the condensed nucleosomes.

Although the Ile 165 in HP1α is crucial for dimerization (30), it is not known whether the corresponding amino acid residue is responsible for HP1γ dimerization. The amino acid sequence alignment shows that Ile 155 in HP1γ is the residue corresponding to Ile165 in HP1α (Supplementary Figure S2). Recombinant HP1γ I155K was purified, and the elution position from size exclusion chromatography was compared to that of wild-type HP1γ. Similar to the HP1α I165K mutant, the apparent molecular size of HP1γ I155K was smaller than that of the wild type (Figure 4B), indicating that the HP1γ I155K was inhibited from self-dimerizing. Interestingly, unlike wild-type HP1γ, HP1γ I155K was not co-precipitated with condensed tetra-nucleosomes by H1.2 (Figure 4C). This result indicates that HP1γ monomer could not bind to H1-condensed H3K9me3 tetra-nucleosomes. Consequently, dimerization of HP1γ through the CSD is necessary for its selective binding to H3K9me3 tetra-nucleosomes in a condensed state. Based on these results, we propose that HP1γ can bind H3K9me3 in tetra-nucleosomes only when the distance between the two H3K9me3 tails in the different core nucleosomes is less than the spatial distance between the two CD in HP1γ dimers.

## DISCUSSION

**Figure 5.**
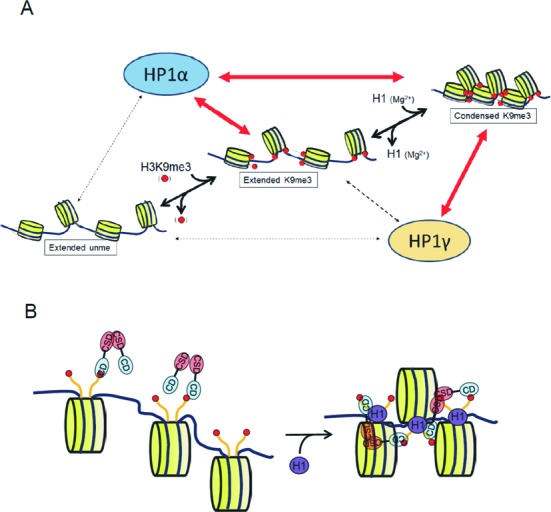
Model for the recognition of H3K9me3 in multi-nucleosomes in different aggregation states. (**A**) Condensation-dependent recognition of H3K9me3 by HP1γ. In contrast to HP1α, HP1γ cannot effectively bind to H3K9me3 in extended nucleosomes, but does bind to H3K9me3 in condensed nucleosomes. The property, however, could not explain the reported localization of HP1γ that preferentially localize to euchromatin and only weakly to the DAPI-dense pericentromeric heterochromatin. The mechanism(s) other than condensation-dependent HP1γ binding could also support the specific localization *in vivo*. (**B**) HP1γ binding to condensed nucleosomes. The distance between the two CD (blue) in dimer HP1γ, which is formed by the interaction between the CSD (red), may not be long enough to reach the two H3K9me3 (red circles) in a core nucleosome. However, the distance between the H3K9me3 residues in two adjacent core nucleosomes is short enough to be reached by the two CD in dimerized HP1γ.

Although the biochemical properties of HP1α and HP1γ are similar to each other (20), distinct *in vivo* functions of HP1α and HP1γ have been reported (14,16–18). The molecular mechanism(s) underlying the distinct *in vivo* functions of HP1 isoforms is still elusive. In the present study, by using nucleosomes as binding substrates, we discovered isoform-specific binding properties of HP1: HP1γ could recognize H3K9me3 in compact nucleosomes but not in extended nucleosomes (Figure 5), whereas HP1α binds in both contexts. We propose that this chromatin structure-dependent binding of HP1γ provides a novel mechanism underlying isoform-specific function *in vivo*.

### Distinct molecular mechanisms of HP1α- and HP1γ-binding to H3K9me3 in nucleosome structure

Mutations in the CSD of HP1γ which disrupt dimerization caused loss of selective binding to H3K9me3 in condensed nucleosomes (Figure 4). These results indicate that dimerization of HP1γ is crucial for binding to H3K9me3 in condensed nucleosomes. This dimerization-dependent selective binding of HP1γ to H3K9me3 tetra-nucleosomes is distinct from that of HP1α, in which recognition of H3K9me3 does not require dimerization (8). Because HR is a flexible, unstructured region (7) and the HR of HP1α extends ten amino acid residues longer than that of HP1γ, we speculate that the distance between the two CD in dimerized HP1γ may not be long enough to recognize two H3K9me3 in one nucleosome core region at the same time (Figure 5B), despite the dynamic mobility of the histone tail (45). The condensation of tetra-nucleosomes might make the H3K9me3 sites in two adjacent nucleosome core particles close enough to be accessed by the dimer HP1γ (Figure 5B). This bivalent binding by the two CD due to dimer formation may increase the apparent affinity to H3K9me3 in condensed tetra-nucleosomes, which allows the low affinity of a single CD to the H3K9me3 to be overcome (Supplementary Figure S1C).

It has been reported that the CD of the yeast HP1 orthologue and of human HP1α undergo dimerization (9,10). On the other hand, the CD–CD interaction was not detected in HP1β by NMR measurement (46). Thus, the ability of the CD to dimerize is somewhat controversial. We could not detect selective binding of HP1γ CD alone to H3K9me3 tetra-nucleosomes in a condensed state (Figure 4), suggesting that, in line with our hypothesis, the CD could not form a dimer by itself.

In a previous study, we have reported that the relatively weak binding affinity of the CD of HP1α to H3K9me3, of which the dissociation constant is 16 μM (8), is underpinned by the DNA-binding activity of the HR when nucleosomes were used as binding substrates (8). The DNA-binding activity of HP1γ was lower than our detection limit (Supplementary Figure S3). Chimeric HP1 with the HP1α-derived HR specifically bound to H3K9me3 in extended tetra-nucleosomes (Figure 1F and G); however, the DNA binding activity of chimeric protein was similarly negligible as to that of HP1γ (Supplementary Figure S3). Thus, isoform-specific HP1 binding activity to extended nucleosomes cannot be simply explained by the DNA binding activity of HR. In contrast, not the CD itself, but the CSD deleted HP1γ was able to bind to both DNA and extended H3K9me3 tetra-nucleosomes (Supplementary Figure S4B and C). Therefore, similar to the case of HP1α, balance between the DNA-binding activity by the hinge region, repressive activity of the CSD, and the H3K9me3-binding via CD could determine the HP1γ binding to extended nucleosomes.

It has been reported that small changes in the linker DNA length affect the rotational relationship between adjacent nucleosomes (47), therefore the linker length may also contribute for the selective binding of HP1. The effect of the linker length on the binding activity of HP1 could be elucidated by future experiments.

### Possible *in vitro* function of HP1γ in nuclei

HP1γ recognized H3K9me3 in histone tails only in condensed nucleosomes (Figures 2 and 3). This observation is supported by a recent report that the H3 tail is dynamic and recognizable by binding proteins even in highly condensed nucleosomes by NMR measurement (48). Thus, induction of nucleosome condensation may not disturb the binding of HP1γ to H3K9me3 in nucleosomes.

HP1α is known to promote chromatin condensation (49). In this study, however, we have shown that HP1γ could not selectively bind to extended nucleosomes, but only to condensed nucleosomes containing H3K9me3 (Figures 3 and 4). HP1γ may contribute to maintaining the heterochromatin status induced by other factors such as H1 and HP1α. This unique property of HP1γ is important in the distinct *in vivo* function compared to that of HP1α.

The aggregation of nucleosomes depends on intrinsic and protein-mediated condensation pathway (50). The nucleosome density, modification states, and concentration of cation are involved in the intrinsic pathway (32,33,50–52). In the presence of 3 mM MgCl_2_, HP1γ recognized the H3K9me3 in the tetra-nucleosomes (Figure 2A), however, could not significantly bind to H3K9me3 mono-nucleosomes (Figure 2D and E), indicating that intra-nucleosome interaction is crucial for the recognition of HP1γ. Concerning protein-mediated condensation, we have tested a physiological factor, histone H1, which also reported to induce nucleosome condensation. As expected, all the isoforms of H1 tested allowed HP1γ to bind selectively to H3K9me3 tetra-nucleosomes. *In vivo*, one of the isoforms H1.2 was co-localized with DAPI-dense regions and HP1γ (Figure 3). Since H1 purified from calf thymus cannot bind to HP1γ (53), it is unlikely that H1.2 directly tethered HP1γ to the heterochromatin. HP1γ was localized to the heterochromatin possibly by recognizing H3K9me3 in DAPI-dense regions (Figure 3D and E). The reason why H1.5 was not co-localized with HP1γ is elusive. Since H1.5 did not co-localize with DAPI-dense regions, one possibility is that HP1γ is not able to co-exist at regions where H1.5 is accumulated. We propose that it is not the localization of H1 isoforms itself, but rather the stabilization of heterochromatin by H1, that may be the driving force for HP1γ to occupy specific subnuclear regions. HP1γ thus shows a more complicated localization in nuclei compared to HP1α, which is localized simply at heterochromatin (7).

Enrichment of HP1γ outside of DAPI-dense heterochromatin could be regulated by unknown mechanisms, involving aspects of chromatin structure outside of histone H1 isoforms. The molecular mechanisms of euchromatic HP1γ enrichment could be elucidated in further work.

Malignant-brain-tumor (MBT) protein, L3MBTL1, is reported to be a component of a complex that also includes HP1γ (54). As the MBT domain of L3MBTL1 possesses chromatin compaction activity in a histone methylation-dependent manner (54), it is possible that HP1γ participates in maintaining heterochromatin as a member of the complex. TIN2, a core component of shelterin, which is a complex required for the protection and replication of chromosome ends, also specifically binds to HP1γ (55). These and other HP1 isoform-specific binding partners may contribute to apparent discrepancies between *in vitro* and *in vivo* HP1γ behavior.

## Supplementary Material

SUPPLEMENTARY DATA
