# Supplementary material for: Nucleosome compaction facilitates HP1γ binding to methylated H3K9
Source: Nucleic Acids Res. 2015 Aug 28;43(21):10200–12. doi: 10.1093/nar/gkv841 (PMC4666388; doi:10.1093/nar/gkv841)
Supplement: SUPPLEMENTARY DATA [file supp_43_21_10200__index.html]

Nucleosome compaction facilitates HP1γ binding to methylated H3K9 — SUPPLEMENTARY DATA 

# Nucleosome compaction facilitates HP1γ binding to methylated H3K9

## SUPPLEMENTARY DATA

- SUPPLEMENTARY DATA
