# Supplementary material for: Nucleosome compaction facilitates HP1γ binding to methylated H3K9
Source: Nucleic Acids Res. 2015 Aug 28;43(21):10200–12. doi: 10.1093/nar/gkv841 (PMC4666388; doi:10.1093/nar/gkv841)
Supplement: SUPPLEMENTARY DATA [file supp_gkv841_nar-01117-m-2015-File007.pdf]

Supplementary Information

# Supplementary Methods

### *Protein purification*

Truncated H1.3 coding 37-211 (ΔN) and 1-111 amino acids (ΔC) were expressed and purified as described elsewhere (1).

### *The affinity between HP1γ and histone H3N-terminal peptides*

The affinities of HP1γ and the CD domain towards the N-terminal tail peptide (1-19) of H3 were determined by ITC as described previously (2). The N-terminal peptide of H3 (1-19) containing H3cK9me3, in which lysine residue was replaced with aminoethyl cysteine, was prepared as described (2). In brief, histone H3 (1-19) carrying K9C mutation was expressed with SUMO-tag in bacteria. The fusion protein was digested with SENP2 protease, and the peptide was purified with reverse phase chromatography. The unmodified or trimethylated lysine analogue was introduced into the peptide as described (3).

### *Gel shift assays*

GST, GST- HP1γ, GST- HP1γ CD+HR, GST- HP1γ CD, GST-HP1α CD+HR, GST- HP1α CD, and GST-HP1γ-αHR (1, 4, and 8 μM) was mixed with 25 nM of monosomal DNA without linker, of length 147bp, described by Mishima *et al.* (2), in the binding buffer including 100 mM NaCl and the binding activity was determined as described in the Materials and Methods. To examine the interaction between GST-HP1γ and DNA in the presence of $MgCl_2$, 3 mM $MgCl_2$, instead of EDTA, was added in the binding buffer including 100 mM NaCl, and separated in a 0.7% agarose gel in 45 mM Tris-borate, and 3 mM $MgCl_2$.

### *$MgCl_2$-dependent nucleosome precipitation*

Nucleosomes were precipitated by centrifugation in the presence of $MgCl_2$ (4, 5). In brief, 40 ng DNA/μl tetra-nucleosomes or mono-nucleosomes in buffer B comprising 2.5 mM NaCl, 0.25 mM EDTA, 10 mM Tris-HCl, pH 7.8 were mixed with an equal volume of buffer B containing $MgCl_2$ to make the indicated final $MgCl_2$ concentrations. After the incubation at room temperature for 10 min, samples were centrifuged at 10,000 x g for 10 min at room temperature. The absorbance at 260 nm of the supernatant fraction, which was not precipitated, was determined in a Nanodrop (Thermo Fisher Scientific, Inc).

### *Gel filtration*

Purified HP1 without GST tag was separated by size exclusion chromatography, as described in Materials and Methods, with slight modification. The buffer used contained either 0.2 mM EDTA or 3 mM $MgCl_2$.

### *$MgCl_2$-dependent binding of HP1γ to tetra-nucleosomes*

To analyze the interaction between HP1γ and reconstituted nucleosomes, 0.2 nM HP1γ and 90 nM of tetra-nucleosomes (amount converted to that of a nucleosome particle with a histone octamer) were mixed in 10 μl of binding buffer containing 6 mM $MgCl_2$, incubated at room temperature for 30 min, and then centrifuged at 10,000 x g for 10 min at 4°C. The supernatant and precipitated fractions were separately electrophoresed in an 18% SDS-polyacrylamide gel. The protein bands were transferred onto a nitrocellulose membrane, and the HP1γ and H4 were immuno-detected with the specific antibodies.

### *Pull down assay determining nucleosome-binding activity of HP1α*

Pull down assay was performed as described in Materials and Methods. Two or four pmol (amount converted to that of a nucleosome particle with a histone octamer) of reconstituted nucleosomes were incubated with 40 or 80 pmol of GST- HP1α-bound GSH-Sepharose, unless otherwise mentioned.

### *H1-dependent nucleosome precipitation*

Tetra-nucleosomes (0.2 μM, amount converted to that of a nucleosome particle with a histone octamer) were incubated with 0.8 μM H1 in the binding buffer, and centrifuged at 10,000 x g for 1 min at 4°C. As a control, 0.5 μM HP1γ, I155K, or the CD, or 0.8 μM H1 alone was centrifuged. The supernatant and precipitated fractions were electrophoresed in an 18% SDS-polyacrylamide gel, and stained with Lumitein.

### *Pull-down assay in the presence of H1*

Pull-down assays were performed as described in the Materials and Methods, except for fixed amount of H1 (four molar excess amount converted to that of a nucleosome particle with a histone octamer) was added to the reaction mixture.

## Legends for Supplementary Figures

**Supplementary Figure S1. Affinity of HP1γ to the N-terminal peptide of H3.**

ITC titration curves of full-length HP1γ (FL) (**A**) and the CD of HP1γ coding (1-75) (CD) **(B)** towards the N-terminal peptide of histone H3 (1-19) containing lysine analogue at K9 with tri-methylated (H3cK9me3) (left panel) or unmethylated (H3cK9me0) (right panels) are shown. **(C)** The dissociation constants are calculated by fitting the data using the ITC data analysis module of Origin 7.0 (OriginLab, Northampton, MA). The dissociation constants are shown in μM. Measurements below the detection limit are indicated as b.d. The dissociation constants for HP1α (*) are taken from a previous report (2).

**Supplementary Figure S2. HP1α, HP1γ, and H1 isoforms used in the present study.**

**(A)** Amino acid sequences of human HP1α and HP1γ aligned with ClustalW (http://clustalw.ddbj.nig.ac.jp/). The CD, HR, and CSD are indicated in light blue, dark blue, and red, respectively. Identical amino acids are indicated as asterisks below the alignment. Colon and dot indicate similar and less similar amino acids, respectively. Ile 165 in HP1α, which is reported to be crucial for dimerization (6), is enclosed with red square. Ile 155 in HP1γ is the corresponding residue to the Ile 155 in HP1α. **(B)** SDS-polyacrylamide gel electrophoresis of purified HP1α, HP1γ, and H1 isoforms. Recombinant GST, GST-tagged wild-type, truncated and mutant HP1α, HP1γ, and their chimera HP1 (upper left panel), HP1α, HP1γ, HP1α I165K, HP1γ I155K, and HP1γ(1-75) (upper right panel), wild type H1.2, H1.3, H1.4, and H1.5 (lower left panel), and the N- and C-terminal truncated H1.3 (lower right panel) (0.5 μg each) were separated in 18% SDS-polyacrylamide gels, and stained with Coomassie Brilliant Blue (CBB). Molecular size markers are indicated at left of each panel.

**Supplementary Figure S3. DNA binding activity of GST-HP1γ and GST-HP1α.**

**(A)** Naked DNA was incubated without protein (lane 1), with GST-HP1α (lanes 2-4), GST- HP1γ (lanes 5-7), GST-HP1γ-αHR (lanes 8-10) or GST (lanes 11-13). To DNA, 40 (lanes 2, 5, 8 and 11), 160 (lanes 3, 6, 9 and 12), or 320 (lanes 4, 7, 10 and 13) molar excess of protein was added, and the binding activity was examined as described in **Figure 3A. (B)** Naked DNA was incubated without (lane 1) or with GST-HP1γ (lanes 2-4). To the DNA, 40 (lane 2), 160 (lane 3), or

320 (lane 4) molar excess of GST-HP1γ was added in the presence of 3 mM $MgCl_2$, and the reaction mixture was separated in a 0.7% agarose containing 45 mM Tris-borate, and 3 mM $MgCl_2$. Arrowhead and bracket indicate the position of free DNA and shifted DNA, respectively.

**Supplementary Figure S4. The CSD of GST-HP1γ negatively regulates the binding to DNA.**

(**A**) Schematic illustration of truncated GST-HP1γ. (**B**) The binding of tetra-nucleosomes to the GST-HP1γ deleted the HR and/or the CSD was determined as described in **Figure 1C**. The GSH-Sepharose bound with the truncated GST-HP1γ was incubated with unmethylated H3 (unme) or H3K9me3 (K9me3) tetra-nucleosomes (left panel). The relative amounts of core histones recovered in the bound fraction over the input (%) were calculated and shown as mean ± S. E. (n=3) (right panel). (**C**) Naked DNA was incubated without protein (lane 1), with GST-HP1αFL (lanes 2-4), GST-HP1αCD+HR (lanes 5-7), GST-HP1αCD (lanes 8-10), GST-HP1γ (lanes 11-13), GST-HP1γCD+HR (lanes 14-16), or GST-HP1γCD (lanes 17-19). To the DNA, 40 (lanes 2, 5, 8, 11, 14 and 17), 160 (lanes 3, 6, 9, 12, 15 and 18), or 320 (lanes 4, 7, 10, 13, 16 and 19) molar excess of protein was added, and the binding activity was determined as described in **Figure 3A.** Arrowhead and bracket indicate the position of free DNA and shifted DNA, respectively.

**Supplementary Figure S5. Effect of $MgCl_2$ concentration on the aggregation state of tetra- and mono-nucleosomes.**

Unmethylated H3 (open circles) and H3K9me3 (closed circles) tetra- (**A**), or mono-nucleosomes (**B**) were incubated with indicated concentrations of $MgCl_2$, and then centrifuged. The absorbance at 260 nm of the input and supernatant fractions was determined, and ratio (%) of tetra- and mono-nucleosomes in the supernatant to the input is shown as mean ± S. E. (n=3).

**Supplementary Figure S6. Effect of $MgCl_2$ on the elution profile of HP1γ on gel filtration.**

HP1γ whose GST-tag was removed was loaded onto a Superdex 200 column (1 x 30 cm), equilibrated with a buffer containing either 0.2 mM EDTA (solid line) or presence of 3 mM $MgCl_2$ (dotted line). Absorbance at 280 nm was monitored.

**Supplementary Figure S7. $MgCl_2$ dependent interaction between HP1γ and tetra-nucleosomes.**

Unmethylated H3 (lanes 1, 2, 5, and 6) or H3K9me3 (lanes 3, 4, 7, and 8) tetra-nucleosomes were incubated with HP1γ, in the reaction buffer containing 50 mM NaCl, either in the presence of 6 mM (lanes 1-4) or in the absence of $MgCl_2$ (lanes 5-8). After incubation, mixtures were centrifuged and then the supernatant (S) and precipitated (P) fractions were separately recovered. Histone H4 and HP1γ in the fractions were detected with Western blotting.

**Supplementary Figure S8. Binding activity of GST-HP1α towards H3K9me3 tetra-nucleosomes in the presence or absence of $MgCl_2$.**

Unmethylated or H3K9me3 tetra-nucleosomes, were incubated with GST-HP1α bound to GSH Sepharose, as described in either Figure 1C (in the absence of $MgCl_2$) or Figure 2A, in the presence of 3 mM $MgCl_2$ and 50 mM NaCl. (**A**) Two pmol of reconstituted nucleosomes were incubated with 80 pmol of GST- HP1γ-bound GSH-Sepharose. The data for binding activity of HP1α in the absence of $MgCl_2$ are taken from previous report (2) (**B**) Four pmol of reconstituted nucleosomes were incubated with 40 pmol of GST-HP1γ-bound GSH-Sepharose. The binding activity was quantitated as in Figure 1C. Means ± S. E. (n=3) are shown.

**Supplementary Figure S9. Precipitation of tetra-nucleosomes in the presence of H1 isoforms.**

Unmethylated H3 (unme, lanes 1, 2, 5, 6, 9, 10, 13, 14, 17 and 18) or H3K9me3 (K9me3, lanes 3, 4, 7, 8, 11, 12, 15, 16, 19 and 20) tetra-nucleosomes were incubated without (lane 1-4), or with H1.2 (lanes 5-8), H1.3 (lanes 9-12), H1.4 (lanes 13-16), or H1.5 (lanes 17-20). After the incubation, the reaction mixtures were centrifuged, and supernatant (S) and precipitated (P) fractions were separately recovered, and then electrophoresed in an 18% SDS-polyacrylamide gel. Arrow and bracket indicate H1 and core histones, respectively. Molecular size markers are shown at the left of the gel (kDa).

**Supplementary Figure S10. HP1γ or/and H1 does not form aggregation.**

HP1γ (WT) (lanes 1 and 2), HP1γ I155K (I155K) (lanes 3 and 4), HP1γ CD (lanes 5 and 6) (0.5 μM), or 0.8 μM of H1.2 (lanes 7 and 8), H1.3 (lanes 9 and 10), H1.4 (lanes 11 and 12), or H1.5 (lanes 13 and 14) **(A)**, and a mixture

containing HP1γ (0.5 µM) and H1 isoforms (0.8 µM), H1.2 (lanes 1 and 2), H1.3 (lanes 3 and 4), H1.4 (lanes 5 and 6), or H1.5 (lanes 7 and 8) **(B)** were centrifuged, supernatant (S) and precipitated (P) fractions were separately recovered, and then analyzed as in **Supplementary Figure S9**. Arrows and arrowheads indicate H1 isoforms and HP1γ, respectively. Molecular size markers (kDa) are shown at the left of images.

**Supplementary Figure S11. Binding of GST-HP1γ to H3K9me3 tetra-nucleosomes condensed by H1 isoforms.**
GST-HP1γ binding to tetra-nucleosomes condensed by H1 isoforms were examined as in **Figure 1C**. GSH-Sepharose, on which GST-HP1γ were anchored, was incubated with unmethylated H3 (unme) or H3K9me3 (K9me3) tetra-nucleosomes. After incubation, input (I), unbound (U), wash (W), and pulled down bound (B) fractions for H1.2, H1.3, H1.4, and H1.5 were separated. Core histones are indicated by brackets (**A**). The relative amounts of core histones recovered in the bound fraction over the input (%) were calculated and shown as mean ± S. E. (n=3) (**B**).

**Supplementary Figure S12. Binding of GST-HP1γ with tetra-nucleosomes in the presence of truncated H1.3.**
**(A)** Schematic illustration of full-length (FL), the N-terminal deleted H1.3 (37-221) (ΔN), and C-terminal deleted H1.3 ΔC (1-111). Globular domains are indicated by ellipses. **(B)** Binding of truncated H1.3 to tetra-nucleosomes. Unmethylated H3 (unme, upper panel) or H3K9m3 (K9me3, lower panel) tetra-nucleosomes were incubated without H1 (lanes 1 and 10) or with H1.3 ΔN (ΔN) (lanes 2-5 and 11-14), or H1.3 ΔC (ΔC) (lanes 6-9 and 15-18). To 0.2 pmol of histone octamer, a half (lanes 2, 6, 11 and 15), equal (lanes 3, 7, 12 and 16), two-fold (lanes 4, 8, 13 and 17), or four-fold (lanes 5, 9, 14 and 18) molar excess of truncated H1.3 was added and electrophoresed. Tetra-nucleosomes and H1-bound fractions of tetra-nucleosomes are indicated by arrowheads and brackets, respectively. **(C)** Effect of truncated H1.3 on the binding of HP1γ to the tetra-nucleosomes (left panel). Bound over input (%) was determined and means ± S. E. (n=3) are shown.

**Supplementary Figure S13. Whole gel images for the main figures.**
The regions excised are shown as red boxes. In panels for Figures 1A, 1C, 1G,

2A, 2C, 2E, 3B, 4A, and 4C, the input (one-fourth of each) (I), unbound (U), wash (W), and bound (B) fractions were electrophoresed. For the binding experiments, unmethylated H3 (unme) or H3K9me3 (K9me3) tetra-nucleosomes were used. The positions of GST, H3, H4, HP1α, HP1γ, and H1 isoforms are indicated in each corresponding images.

## REFERENCES


1, Takeshima, H., Suetake, I., and Tajima, S. (2008). Mouse Dnmt3a preferentially methylates linker DNA and is inhibited by histone H1. *J. Mol. Biol.,* **383**, 810-821.

2, Mishima, Y., Watanabe, M., Kawakami, T., Jayasinghe, C. D., Otani, J., Kikugawa, Y., Shirakawa, M., Kimura, H., Nishimura, O., Aimoto, Tajima, S., and Suetake, I. (2013) Hinge and chromoshadow of HP1α participate in recognition of K9 methylated histone H3 in nucleosomes. *J. Mol. Biol.,* **425**, 54-70.

3, Simon, M. D. (2010) Installation of site-specific methylation into histones using methyl lysine analogs. *Curr. Protoc. Mol. Biol. Chapt* 21: unit 21.18, pp. 1-10, John Wiley and Sons, Inc.

4, Schwarz, P. M and Hansen, J. C. (1994) Formation and Stability of Higher Order Chromatin Structures. *J. Biol. Chem.,* **269,** 16284-16289.

5, Blacketer, M. J., Feely, S. J., and Shogren-Knaak, M. A. (2010) Nucleosome Interactions and Stability in an Ordered Nucleosome Array Model System. *J. Biol. Chem.,* **285**, 34597-34607.

6, Lechner, M. S., Schultz, D. C., Negorev, D., Maul, G. G., and Rauscher, F. J. 3rd. (2005) The mammalian heterochromatin protein 1 binds diverse nuclear proteins through a common motif that targets the chromoshadow domain. *Biochem. Biophys. Res. Commun.,* **331**, 929-937.

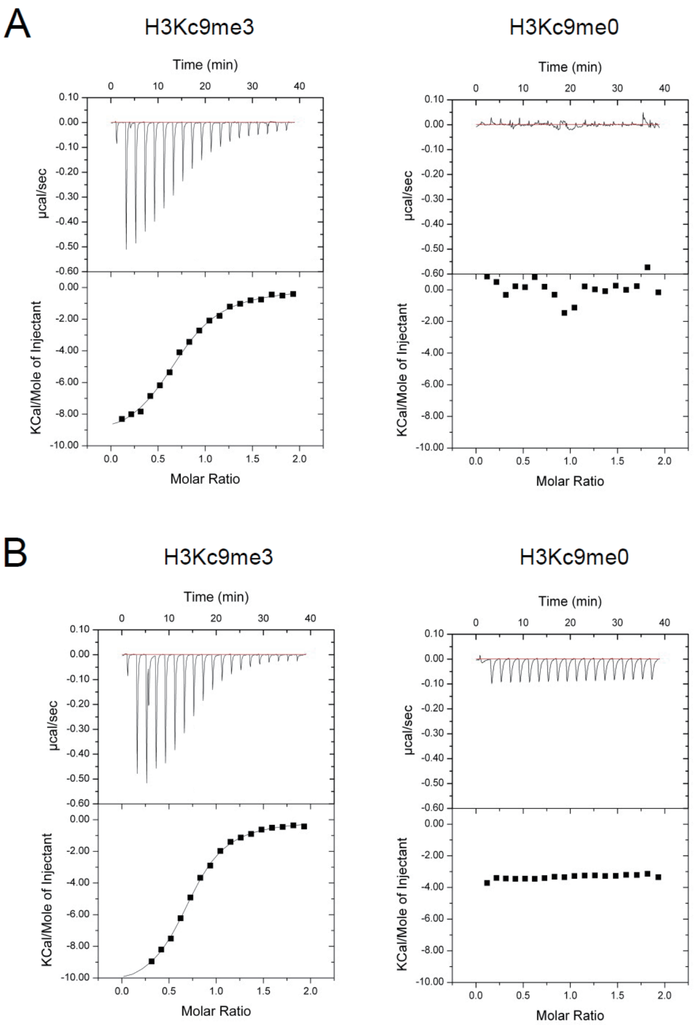


C

| | | H3Kc9me0 | H3Kc9me3 |
|---|---|---|---|
| HP1α | FL | b.d. | 12* |
| | CD | b.d. | 16* |
| HP1γ | FL | b.d. | 2.5 |
| | CD | b.d. | 3.9 |

Supplementary Figure S1

A

```
HP1α     1 MGKKTKRTADSSSSEDEEEYVVEKVLDRRVVKGQVEYLLKWKGFSEEHNTWEPEKNLDCP 60
HP1γ     1 MGKKQNGKSKKVEEAEPEEFVVEKVLDRRVVNGKVEYFLKWKGFTDADNTWEPEENLDCP 60
           **** : .:.. .. : **:***********:*:***:******:: .******:*****
                                              chromo domain (CD)

HP1α    61 ELISEFMKKYKKMKEGENNKPREKSESNKRKSNFSNSADDIKSKKKREQSNDIARGFERG 120
HP1γ    61 ELIEAFLNSQKAGKE---------KDGTKRKSLSDSESDDSKSKKKRD-AADKPRGFARG 110
           ***. *::. *  **         .:..****  ...:** ******: : * .*** **
                                              hinge region (HR)

HP1α   121 LEPEKIIGATDSCGDLMFLMKWKDTDEADLVLAKEANVKCPQIVIAFYEERLTWHAYPED 180
HP1γ   111 LDPERIIGAIDSSGELMFLMKWKDSDEADLVLAKEANMKCPQIVIAFYEERLTWHSCPED 170
           *:**:**** **.*:*********:************:*****************: ***
                                  chromo shadow domain (CSD)

HP1α   181 AENKEKETAKS 193
HP1γ   171 EAQ-------- 173
           :
```

B

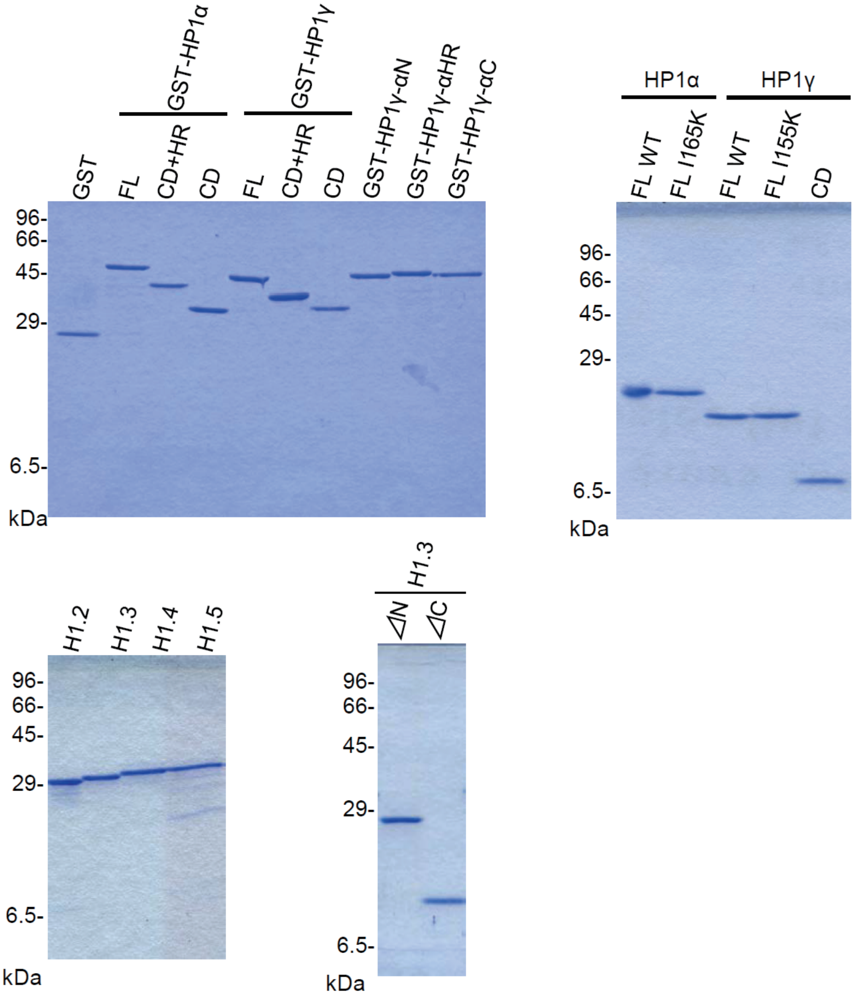


Supplementary Figure S2

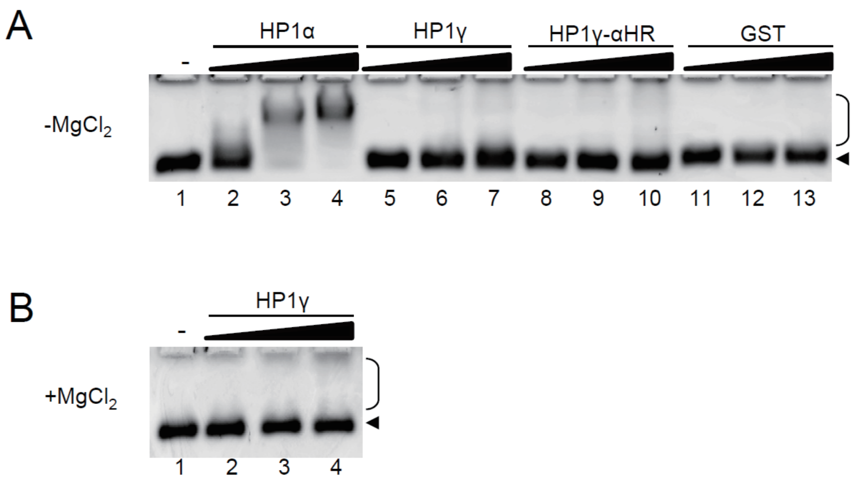


Supplementary Figure S3

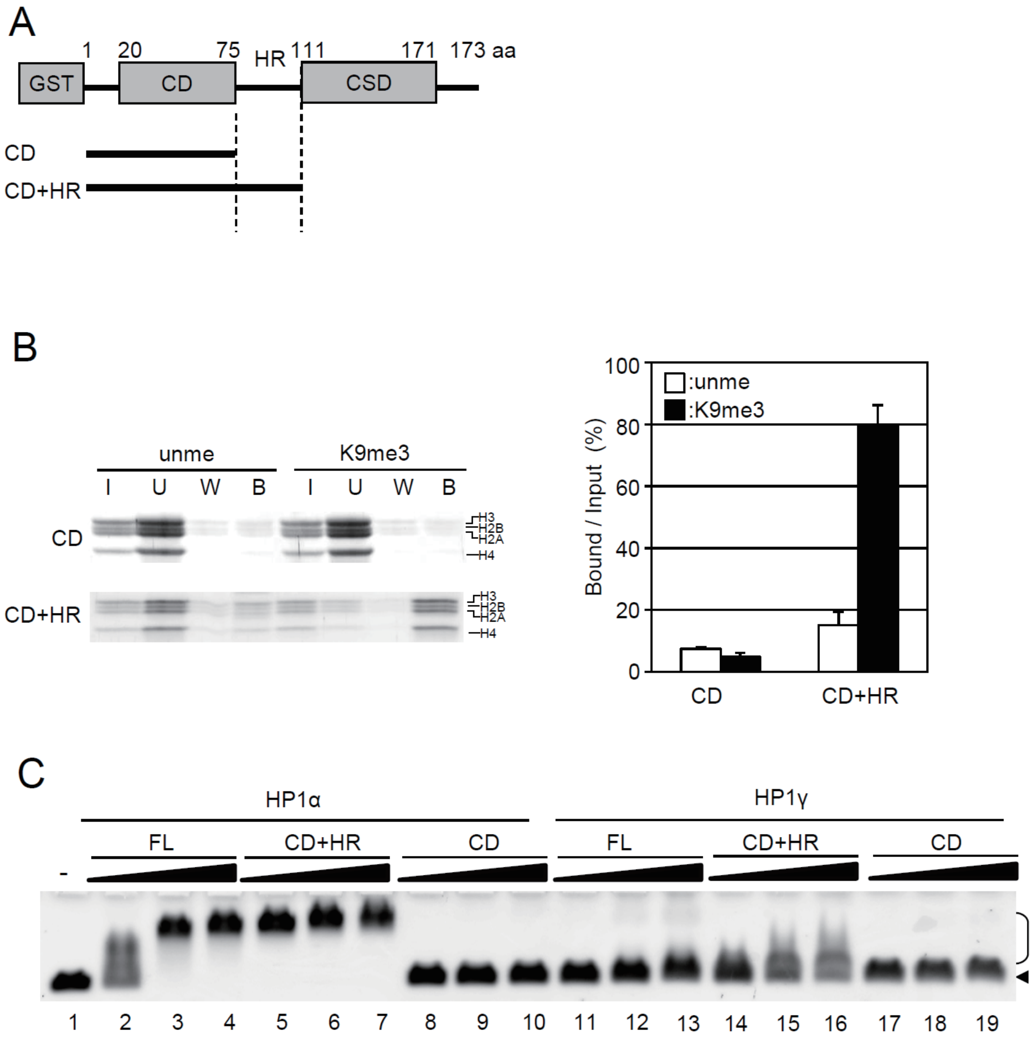


Supplementary Figure S4

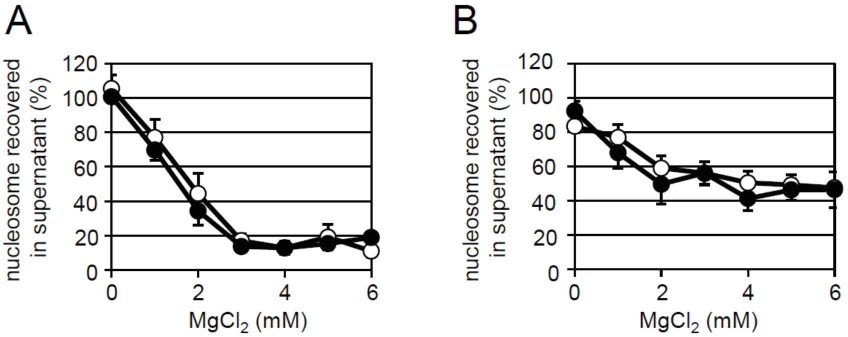


Supplementary Figure S5

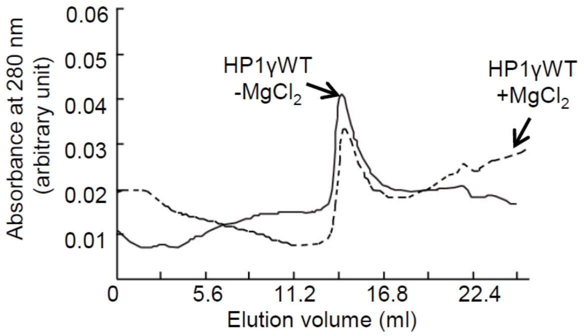


Supplementary Figure S6

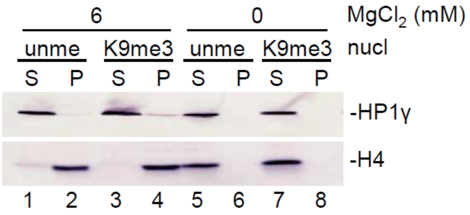


Supplementary Figure S7

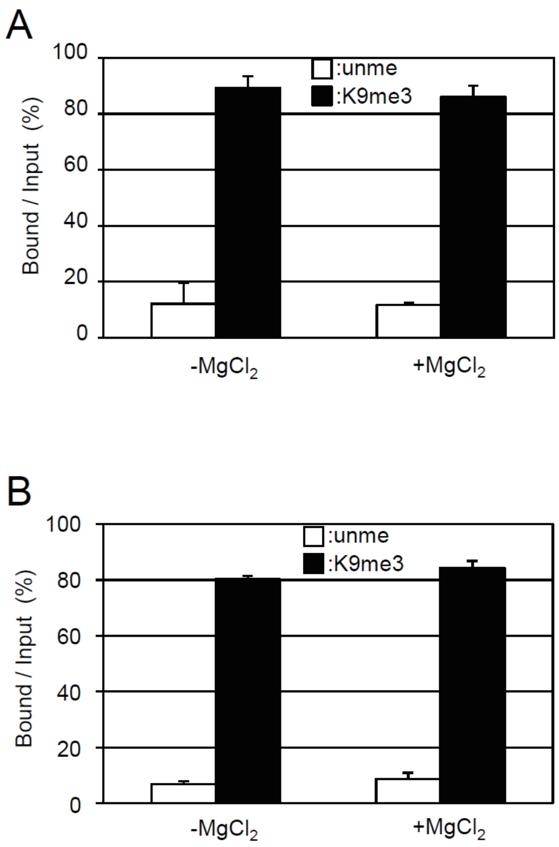


Supplementary Figure S8

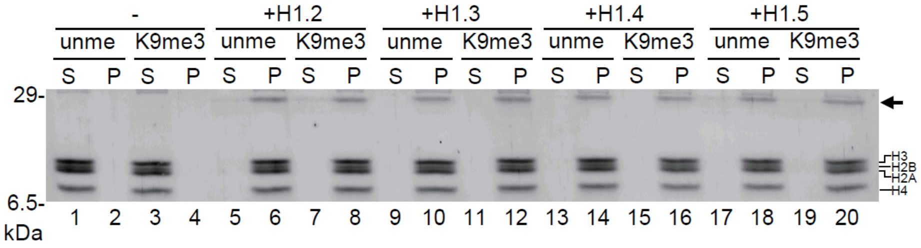


Supplementary Figure S9

A

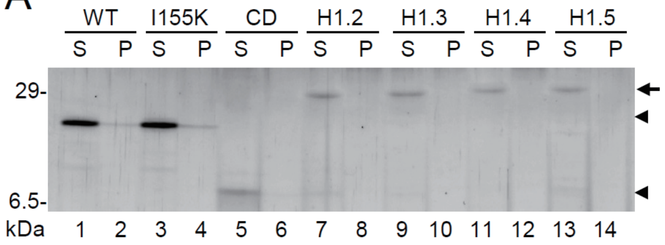


B

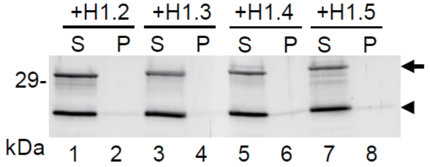


Supplementary Figure S10

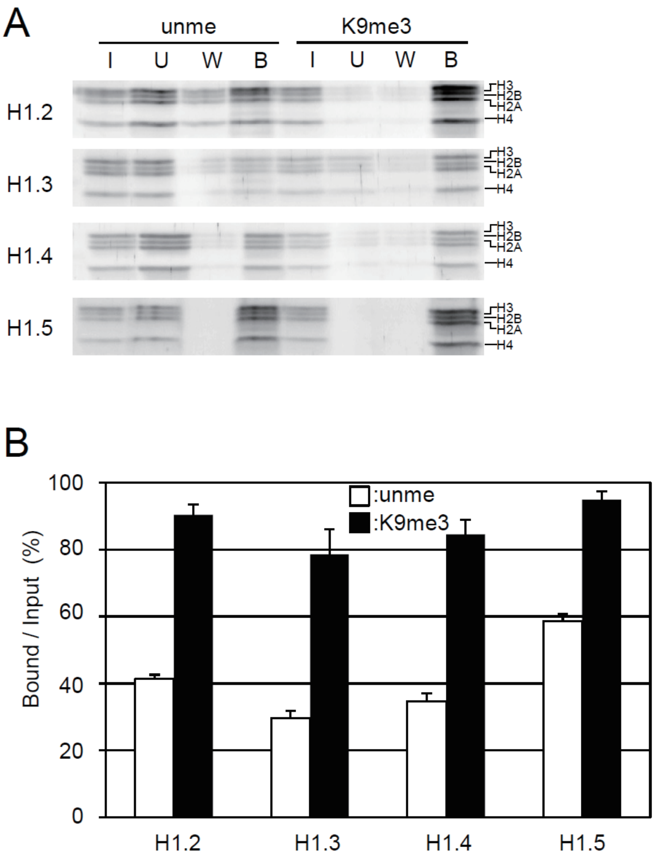


Supplementary Figure S11

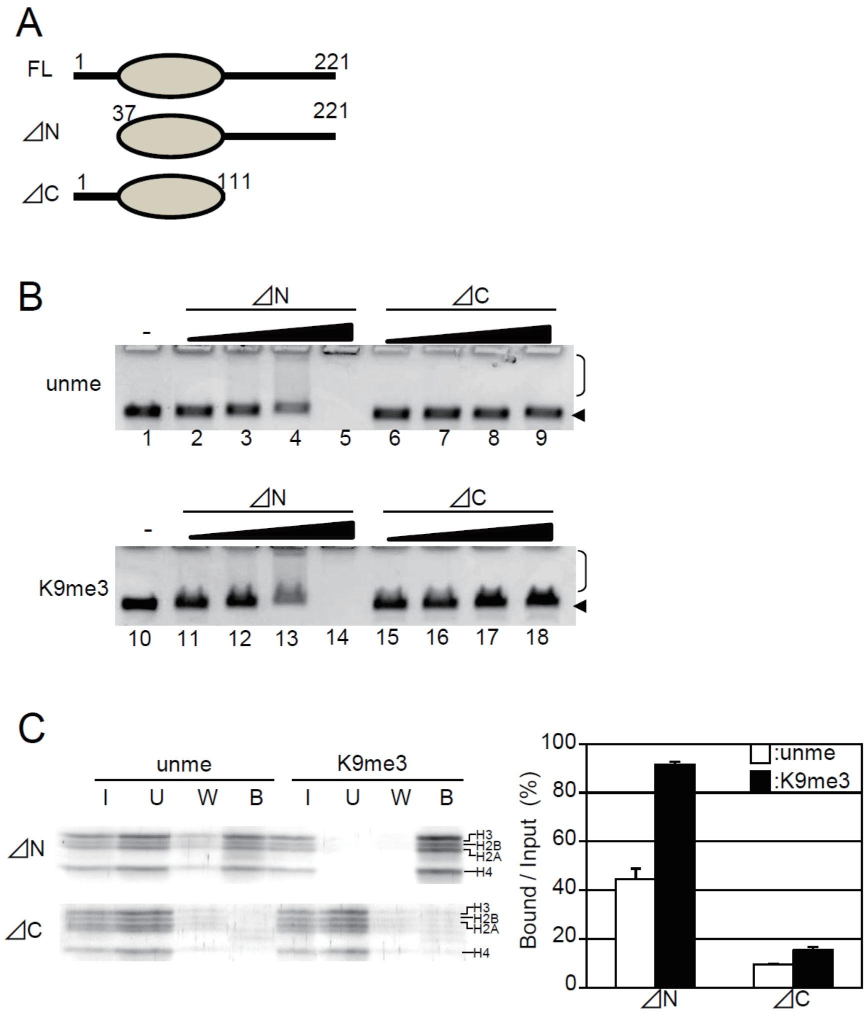


Supplementary Figure S12

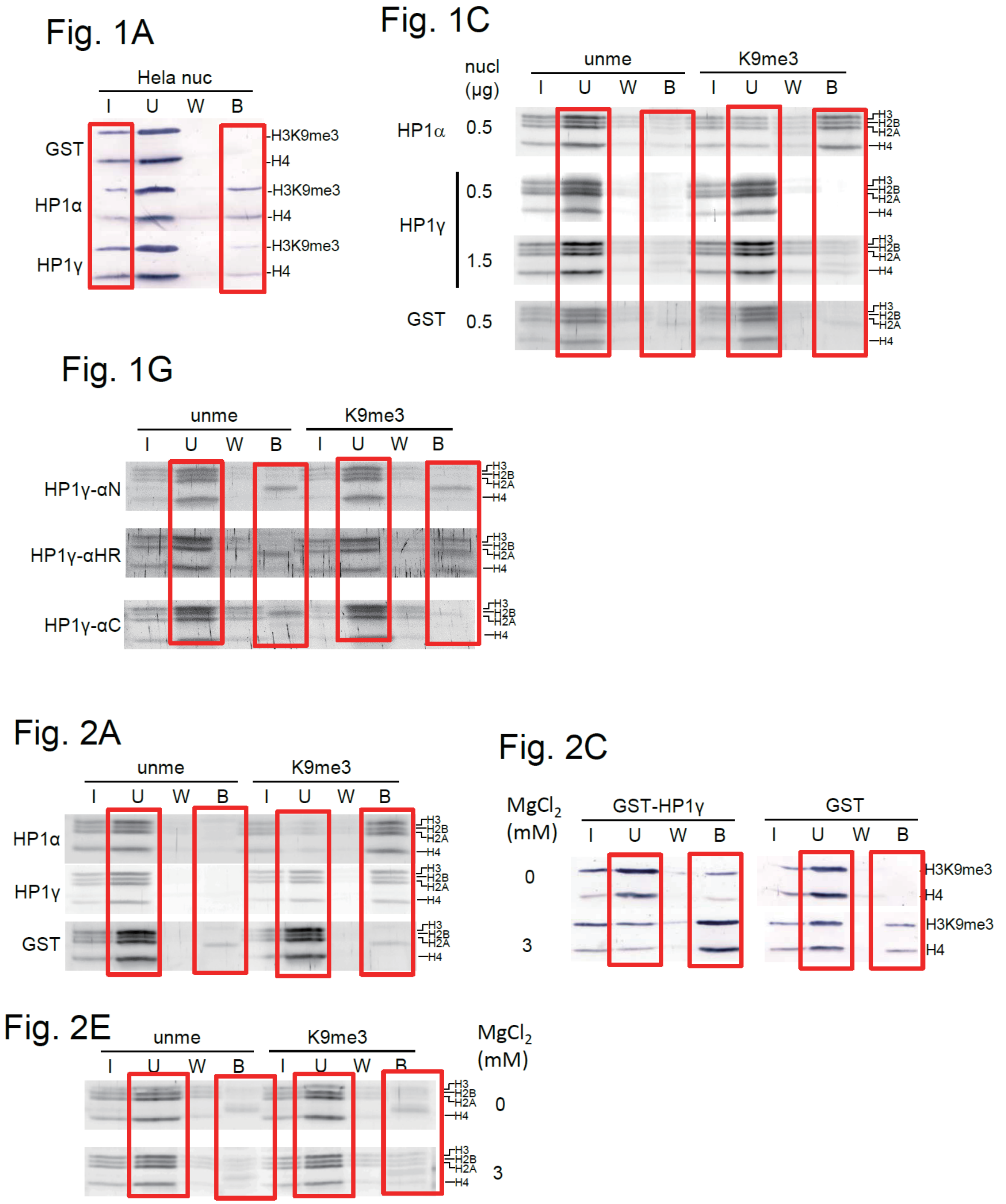


Supplementary Figure S13

## Fig. 3B

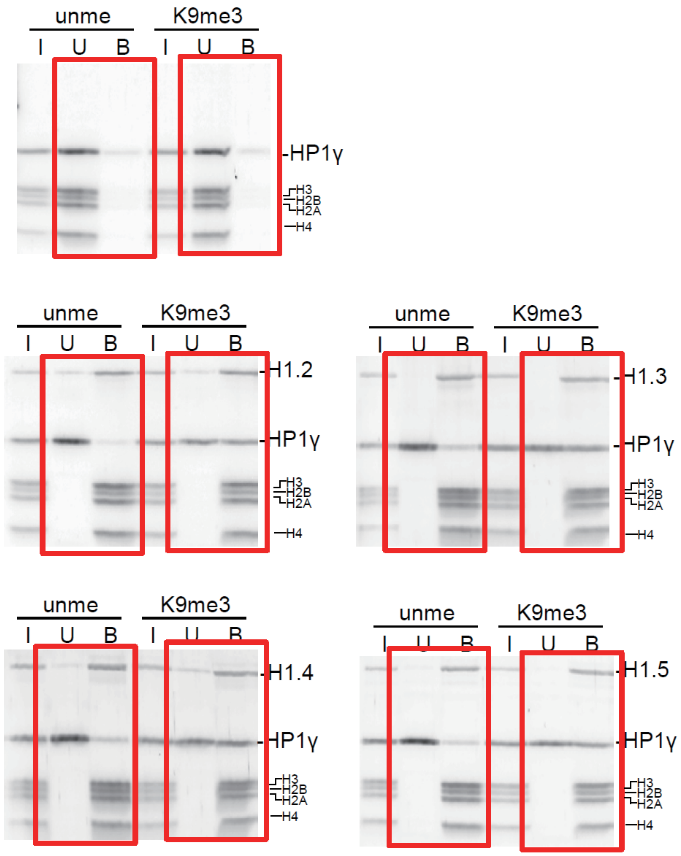


## Fig. 4A

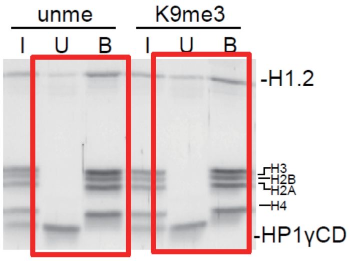


## Fig. 4C

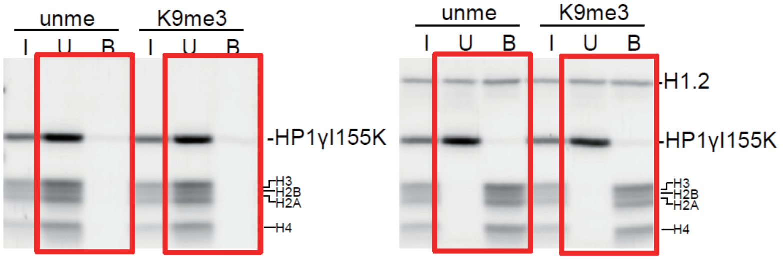


Supplementary Figure S13 (continued)
